# Coordination of MAPK and p53 dynamics in the cellular responses to DNA damage and oxidative stress

**DOI:** 10.15252/msb.202211401

**Published:** 2022-12-06

**Authors:** Ryan L Hanson, Eric Batchelor

**Affiliations:** ^1^ Department of Integrative Biology and Physiology University of Minnesota Minneapolis MN USA; ^2^ Masonic Cancer Center University of Minnesota Minneapolis MN USA

**Keywords:** cell stress responses, dynamics, MAPKs, p53, single cells, Autophagy & Cell Death, Signal Transduction

## Abstract

In response to different cellular stresses, the transcription factor p53 undergoes different dynamics. p53 dynamics, in turn, control cell fate. However, distinct stresses can generate the same p53 dynamics but different cell fate outcomes, suggesting integration of dynamic information from other pathways is important for cell fate regulation. To determine how MAPK activities affect p53‐mediated responses to DNA breaks and oxidative stress, we simultaneously tracked p53 and either ERK, JNK, or p38 activities in single cells. While p53 dynamics were comparable between the stresses, cell fate outcomes were distinct. Combining MAPK dynamics with p53 dynamics was important for distinguishing between the stresses and for generating temporal ordering of cell fate pathways. Furthermore, cross‐talk between MAPKs and p53 controlled the balance between proliferation and cell death. These findings provide insight into how cells integrate signaling pathways with distinct temporal patterns of activity to encode stress specificity and drive different cell fate decisions.

## Graphical abstract

In single pancreatic cells responding to oxidative stress, JNK dynamics regulate an early trigger of cell death, p53 controls a late maintenance of cell cycle arrest, and p38 controls a switch between JNK‐ and p53‐dependent cell fate.

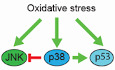

## Introduction

The transcription factor p53 regulates cellular stress responses by regulating the expression of target genes involved in DNA repair, cell cycle arrest, apoptosis, and senescence. In response to stress, p53 is rapidly stabilized through post‐translational modifications that block its degradation by negative regulators including MDM2 (Haupt *et al*, 1997; Kubbutat *et al*, 1997; Shieh *et al*, 1997). The network of positive and negative feedback that regulates p53 stability leads to distinct temporal patterns of p53 expression in response to different forms of cellular stress. The dynamic expression patterns of p53 include undamped oscillations in response to DNA double‐strand breaks (DSBs) (Bar‐Or *et al*, 2000; Lahav *et al*, 2004; Geva‐Zatorsky *et al*, 2006), a single graded pulse in response to UV radiation (Batchelor *et al*, 2011), and monotonically increasing accumulation in response to continuous exposure to chemotherapeutics (Paek *et al*, 2016; Yang *et al*, 2018). p53 dynamics have several functions during cell stress responses, including distinguishing between different stresses (Batchelor *et al*, 2011; Purvis *et al*, 2012; Paek *et al*, 2016), dictating cell fate outcomes (Purvis *et al*, 2012; Chen *et al*, 2016; Paek *et al*, 2016; Yang *et al*, 2018), and diversifying expression of downstream p53 targets (Porter *et al*, 2016; Hafner *et al*, 2017; Hanson *et al*, 2019). However, p53 dynamics can be highly variable, potentially limiting the information encoded by p53 expression dynamics alone. Evidence of this limitation is the observation that p53 dynamics are not discrete, instead existing across a broad spectrum of potential dynamics depending on the cell line, DNA damage dose, and intrinsic repair rate of DNA lesions (Stewart‐Ornstein & Lahav, 2017). How, then, do cells generate distinct cellular stress responses in conditions where p53 dynamics are similar?

Due to variability in the concentrations and activities of signaling molecules in individual cells, recent studies have shown that many pathways can only reliably distinguish approximately two distinct states, such as the presence or absence of a ligand or stimulus (Cohen‐Saidon *et al*, 2009; Bao *et al*, 2010; Cheong *et al*, 2011; Suderman *et al*, 2017). Cells have evolved mechanisms to circumvent such a limitation in information transfer. For example, to increase specificity in the response to TNF concentrations the NF‐κB pathway integrates information from a parallel signaling pathway mediated by ATF2 (Cheong *et al*, 2011). In response to p53‐activating stresses, other parallel signaling pathways, including the mitogen‐activated protein kinases (MAPKs) ERK, JNK, and p38, can be upregulated. Several regulatory interactions between the MAPKs and p53 have been identified (Fuchs *et al*, 1998; She *et al*, 2000), suggesting that cross‐talk between the networks and integration of their signaling activities may be a method to improve specificity in p53‐dependent stress responses.

To determine whether specific features of MAPK dynamics are integrated with p53 dynamics to increase specificity in p53‐dependent stress responses, we first identified two distinct stresses, DNA double‐strand breaks (DSBs) and oxidative stress, that generated similar p53 dynamics but led to different cell fate outcomes. We expressed kinase biosensors of ERK, JNK, or p38 (Regot *et al*, 2014) activity in cell lines expressing fluorescently tagged p53 to simultaneously measure the dynamics of MAPK activities and p53 expression in single cells. Unlike p53, the MAPKs exhibited preferential activation in response to oxidative stress in a dose‐dependent manner. Selective inhibition of the MAPKs led to changes in cell survival, indicating their importance in cell fate determination. Using our single‐cell approach, we found that JNK activity was an important regulator of oxidative stress‐induced cell death; in contrast, p53 dynamics were important for maintaining cell cycle arrest. The stress‐activated kinase p38 functions in the cross‐talk between the JNK and p53, inhibiting JNK activation and upregulating p53 to influence the relative balance between the two distinct cell fate outcomes. These findings highlight how the integration of signals from multiple dynamic networks can specify cell fate determination in response to cellular stress.

## Results

### Cells can drive distinct cell fate outcomes despite similar p53 dynamics

**Figure 1:**
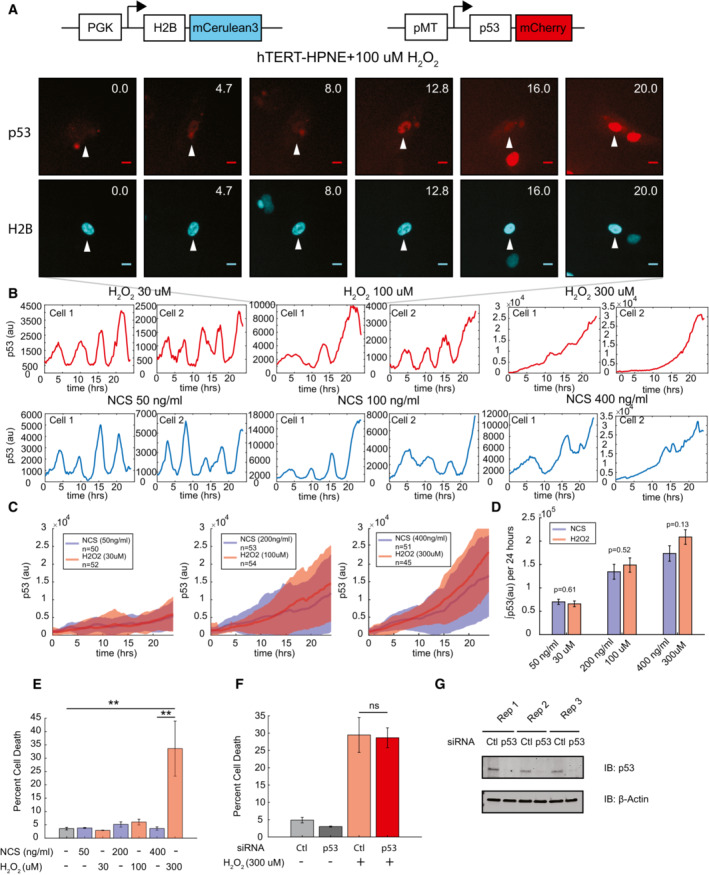
Similar p53 dynamics can drive distinct cell fates p53 expression dynamics were measured in single cells using a fluorescently labeled p53‐mCherry construct in conjunction with an H2B‐mCerulean nuclear marker to define the nucleus of individual cells. Images show representative single cells over time. Arrowhead highlights a single cell over multiple time points. Scale bar = 20 μm.Single‐cell traces for representative cells exposed to increasing doses of H_2_O_2_ (red) or NCS (blue). Dynamics switch from pulses at low doses of damage to rising dynamics at higher doses.Comparison of population averages of single‐cell p53 expression in response to H_2_O_2_ (red) or NCS (blue) across doses. Thick line represents the mean p53 fluorescence intensity with shaded areas representing ± SD. *n* represents the number of individual cells analyzed per condition.Bar graphs comparing integrated p53 expression over the 24‐h range for both NCS and H_2_O_2_ treatment. *P*‐values calculated by one‐way ANOVA. Data represent mean ± SEM based on the number of cells analyzed for each condition (*n*). *n* for each condition is shown in (C).Percentage of cell death was calculated by flow cytometry staining for Annexin V and propidium iodide for indicated doses of NCS and H_2_O_2_ 24‐h post‐treatment. *P*‐values were calculated using one‐way ANOVA for multiple comparisons. Data represent the mean ± SEM for three biological replicates (*n* = 3).Percentage of cell death in the presence or absence of p53 knockdown after 24‐h with 300 μM H_2_O_2_ treatment. Data represent the mean ± SEM for three biological replicates (*n* = 3). Statistics were performed using one‐way ANOVA.Western blot analysis of siRNA knockdown of p53 expression in three biological replicates (*n* = 3). β‐actin serves as an internal loading control. Data information: **P < 0.01, ns = not significant.

To study p53‐dependent cell stress responses in individual, nontransformed cells, we established a clonal cell line expressing a fluorescently labeled H2B‐mCerulean nuclear marker and fluorescently labeled p53‐mCherry in the immortalized nontransformed pancreatic cell line hTERT‐HPNE (Fig 1A), a system that has been previously used to study p53 dynamics in other cell lines (Batchelor *et al*, 2011). We used the system to track p53 levels and cell fate outcomes in response to two stresses, DNA double‐strand breaks generated by the radiomimetic drug neocarzinostatin (NCS) and oxidative stress generated by H_2_O_2_, in single cells. To determine p53 expression dynamics in response to these two stimuli, we treated cells with several doses of NCS and H_2_O_2_ and measured the mean fluorescence intensity of p53‐mCherry over time in the nuclei of individual cells. Similar p53 dynamics were generated in response to the two stresses, which was evident at both the single cell (Fig 1B) and the population levels (Fig 1C). At low doses of stimulus, p53 levels were oscillatory; at higher doses, p53 levels increased steadily. We confirmed that fluorescently tagged p53 accurately reflected the dynamics of endogenous p53 by western blot analysis of high‐dose H_2_O_2_‐treated cells (Appendix Fig S1A). Integrated levels of p53 over the 24‐h time course were quantitatively similar for the two stresses (Fig 1D).

p53 dynamics regulate cell fate in response to DNA damage (Purvis *et al*, 2012; Paek *et al*, 2016; Yang *et al*, 2018). To determine whether cells could induce different cell fate outcomes despite similar p53 dynamics, we compared cell death across NCS and H_2_O_2_ treatment. Using flow cytometry, we found the rate of cell death in hTERT‐HPNE cells treated with H_2_O_2_ was significantly elevated compared with NCS‐treated cells at the highest tested dosages for each stimulus (Fig 1E). These results were supported in our single‐cell imaging studies, as we observed reduced cell viability in response to high‐dose H_2_O_2_ treatment (Appendix Fig S1B). Unexpectedly, although p53 regulates several pro‐apoptotic target genes the increase in cell death we observed in hTERT‐HPNE was independent of p53, as siRNA knockdown of p53 did not reduce cell death compared with a nontargeting control (Fig 1F). Knockdown of p53 was confirmed by western blot analysis (Fig 1G). Similar results were obtained with a second siRNA against p53 (Appendix Fig S1C and D). While p53 expression was dispensable for the regulation of cell death, the diverse roles of p53 suggested it could be acting to upregulate other cell fate response pathways such as DNA repair or cell cycle regulation. Taken together, these results suggest that p53 dynamics alone are insufficient to specify distinct cell fates in response to NCS and H_2_O_2_ and that additional pathways must operate in parallel to generate the differential responses.

### ERK, JNK, and p38 kinases show preferential activation in response to oxidative stress

**Figure 2:**
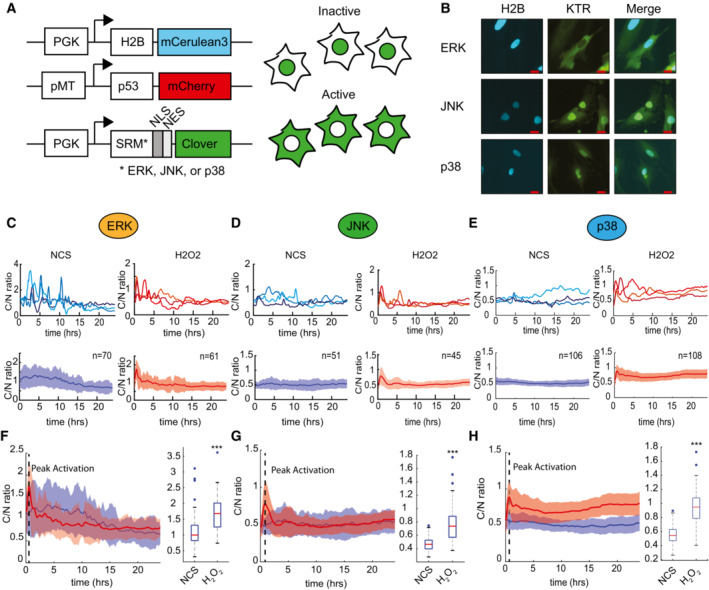
MAPKs exhibit distinct activation profiles between double‐strand breaks and oxidative stress A
Schematic of hTERT‐HPNE cell lines used to assess p53 and MAPK signaling. Each cell line contained an H2B‐mCerulean nuclear marker, p53‐mCherry, and kinase translocation reporter (KTR) for either ERK, JNK, or p38 labeled with mClover. KTR provides a readout of kinase activity based on subcellular localization.B
Images represent localization of KTR for ERK, JNK, or p38 under basal growth conditions. Scales bars = 20 μm.C–E
Kinase activity for ERK (C), JNK (D), or p38 (E) in three representative single cells (upper panels) in response to 400 ng/ml NCS (blue) or 300 μM H_2_O_2_ (red) as measured by C/N ratio. Lower panel shows the mean C/N ratio in response to 400 ng/ml NCS (blue) or 300 μM H_2_O_2_ (red). Thick line represents the mean with shaded area = SD. *N* for each condition and kinase is shown in population average plots.F–H
Comparison of kinase activity measured by C/N ratio for ERK (F), JNK (G), or p38 (H) in response to 400 ng/ml NCS (blue) or 300 μM H_2_O_2_ (red). Thick line represents the mean with shaded area = SD. Box plots compare the C/N ratio at the observed H_2_O_2_‐induced peak (dotted line). Central band represents the median value, with the box bounding the 25^th^–75^th^ percentiles. Whiskers extend to extreme values not considered an outlier (approximately ±2.7σ). *n*, for each condition and kinase, is shown in the above plots and represents the number of individual cells analyzed per condition. Statistics were performed using one‐way ANOVA (***P < 0.001). Data information: ***P < 0.001.

The mitogen‐activated protein kinases ERK, JNK, and p38 are activated in response to oxidative stress (Minamino *et al*, 1999), and activation dynamics have been shown to be important for downstream regulatory responses for ERK (Albeck *et al*, 2013; Ryu *et al*, 2015; Gagliardi *et al*, 2021) and JNK (Miura *et al*, 2018). Therefore, we hypothesized that MAPK dynamics may coordinate with p53 dynamics to control temporal regulation of the oxidative stress response. To simultaneously quantify MAPK activity and p53 expression in single cells in response to H_2_O_2_, we incorporated kinase translocation reporter (KTR) biosensors specific for ERK, JNK, or p38 (Regot *et al*, 2014) into our hTERT‐HPNE cells expressing fluorescently tagged p53 and H2B (Fig 2A). The KTR biosensors report kinase activity by shuttling between the nucleus and cytoplasm based on phosphorylation of key MAPK‐targeted residues. Quantifying the ratiometric expression of the sensor between the two compartments provides a quantitative measure of kinase activity. Under unstressed conditions, both JNK and p38 were largely inactive and their respective sensors were primarily localized in the nucleus (Fig 2B). The ERK biosensor was primarily localized within the cytoplasm in many cells (Fig 2B), likely due to hTERT‐HPNE cells relying on exogenous EGF for growth.

To determine whether activation dynamics of ERK, JNK, or p38 differed between DSBs and oxidative stress, we treated cells with 400 ng/ml NCS or 300 μM H_2_O_2_. We quantified kinase activity by measuring the mean cytoplasmic‐to‐nuclear ratio (C/N ratio) of fluorescence intensity for each biosensor to quantify ERK (Fig 2C), JNK (Fig 2D), or p38 (Fig 2E) activity. Treatment of cells with H_2_O_2_ led to significantly increased activation of all three kinases (Fig 2F–H). Interestingly, we did not observe obvious ERK activation in response to NCS as had previously been reported in MCF7 cells examining ERK and p53 responses (De *et al*, 2020). This may reflect sustained ERK signaling due to EGF in the hTERT‐HPNE media. We confirmed that the kinase reporters accurately reported early activation of the kinases by using western blot analysis to detect active phosphorylated forms for each kinase at 30‐min post‐treatment, near the peak of kinase activation based on the microscopy results (Appendix Fig S2A–C). Like p53, the activation of the kinases was H_2_O_2_‐dose dependent with maximum kinase activation occurring at the lethal 300 μM dose (Appendix Fig S2D–F). Given the increased activation of the MAPKs in response to high‐dose H_2_O_2_ but not NCS, we hypothesized that the dynamics of the kinases may be responsible for generating the observed differences in cell fate between the two stress stimuli.

### Dynamic activation of JNK plays a key role in oxidative stress‐induced cell death

**Figure 3:**
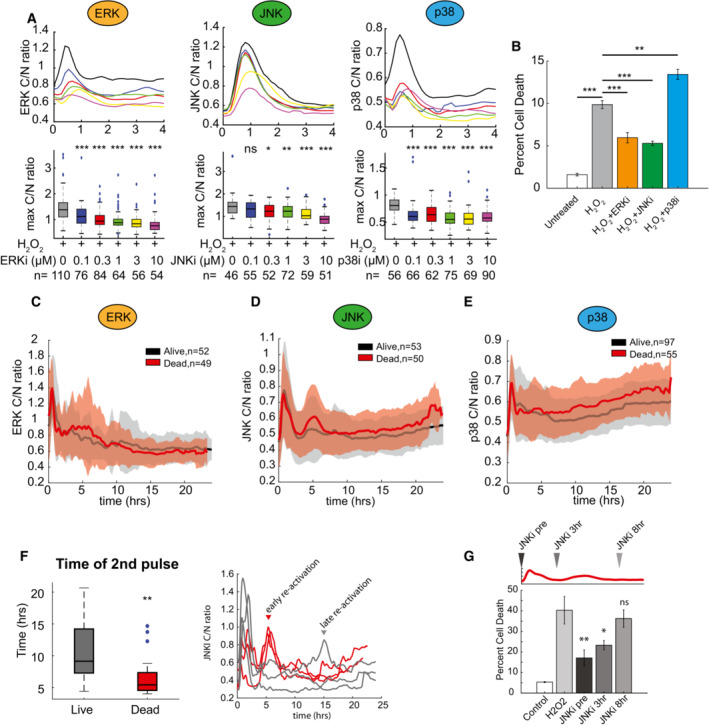
JNK dynamics regulate the induction of cell death A
Upper panel shows population average traces of ERK, JNK, or p38 in response to 300 μM H_2_O_2_ in the presence or absence of specific inhibitors. Lower panels show boxplots with max C/N ratio for each condition. Central band represents the median value, with the box bounding the 25^th^–75^th^ percentiles. Whiskers extend to extreme values not considered an outlier (approximately ±2.7σ). Statistics were performed using one‐way ANOVA (*P < 0.05, **P < 0.01, ***P < 0.001, ns = not significant); *n* represents the number of individual cells analyzed.B
Percentage of cell death 24‐h post‐treatment with 300 μM H_2_O_2_ in the presence or absence of ERK, JNK, or p38 inhibition. Statistics were performed using one‐way ANOVA (**P < 0.01, ***P < 0.001). Data represent mean ± SEM. Results based on three biological replicates (*n* = 3).C–E
Mean kinase activation for ERK (C), JNK (D), or p38 (E) in both live (gray) and dead (red) cells measured over 24‐h in response to H_2_O_2_. Shaded areas = SD. *N* for each condition is shown.F
Left panel has boxplot showing the distribution of timing on the second JNK pulse between surviving and dying cells. Central band represents the median value, with the box bounding the 25^th^–75^th^ percentiles. Whiskers extend to extreme values not considered an outlier (approximately ±2.7σ). Statistics were performed using a 2‐tailed *t*‐test (**P < 0.01). Right plot shows representative single‐cell traces of surviving (gray) cells and dying (red) cells to highlight early JNK re‐activation. Number of analyzed cells for each condition (*n*) is shown in panel (D).G
Percentage of cell death 24‐h post‐H_2_O_2_ treatment in response to different JNK inhibition timings. Upper schematic illustrates the timing of different JNK inhibition regimens. Data represent mean ± SEM based on a minimum of three independent biological replicates (*n* = 3). Statistics were performed by one‐way ANOVA (*P < 0.05, **P < 0.01, ns = not significant). Data information: *P < 0.05, **P < 0.01, ***P < 0.001, ns = not significant.

To determine the role of ERK, JNK, and p38 dynamics in H_2_O_2_‐induced cell death, we first determined conditions under which we could pharmacologically inhibit each kinase. We challenged cells with different doses of ravoxertinib (ERK inhibitor), tanzisertib (JNK inhibitor), or FHPI (p38 inhibitor) and measured peak kinase activation by C/N ratio in response to H_2_O_2_ (Fig 3A). Maximal inhibition of each kinase resulted from a 10 μM dose of respective inhibitor, which was therefore used as the inhibitor concentration in subsequent experiments. We further confirmed this dosage inhibited the activation of known downstream pathways by western blot analysis (Appendix Fig S3A). We next assessed by flow cytometry the impact on cell death upon inhibition of each individual MAPK (Fig 3B). In response to H_2_O_2_ treatment, inhibition of either ERK or JNK led to a reduction in cell death, whereas inhibition of p38 increased the percentage of cell death, indicating that the activity of all three MAPKs affects H_2_O_2_‐induced cell death. Similar trends in cell survival were observed by quantifying cell viability by live‐cell imaging (Appendix Fig S3B) and by using an additional set of pharmacological inhibitors (Appendix Fig S3C).

Dynamic patterns of p53 expression in response to DNA damage are predictive of cell fate outcomes (Paek *et al*, 2016; Yang *et al*, 2018). To determine whether we could identify specific features of MAPK dynamics that are predictive of cell death activation in response to oxidative stress, we imaged cells in response to 300 μM H_2_O_2_ over 24‐h and compared ERK, JNK, or p38 activity in cells that survived treatment versus cells that eventually died (Fig 3C–E). For ERK and JNK activity, we quantified the maximum C/N ratio, timing of activation pulses, duration of pulses, and number of pulses of activity, comparing differences for each characteristic between surviving and dying cells over the 24‐h window (Appendix Fig S3D and E). As p38 activity was largely sustained with few discernible pulses, for p38 we measured the maximum C/N ratio and the timing of the maximum C/N ratio (Appendix Fig S3F). We found that cells with repeated pulses of JNK activation within the first 8 h following H_2_O_2_ treatment showed elevated levels of cell death (Fig 3F). To determine whether the JNK pulses had a biological impact on the induction of cell death, we selectively inhibited JNK activity using tanzisertib at three specific times: just prior to H_2_O_2_ treatment, between the first and second pulse of JNK activity, or after the second pulse of JNK activity (Fig 3G). We found that inhibition of JNK either prior to or 3 h after H_2_O_2_ treatment significantly reduced levels of cell death; however, the inhibition of JNK 8 h after H_2_O_2_ treatment did not affect cell death. These results suggested that early JNK activation in a series of two pulses is important for inducing cell death (Fig 3G).

### H_2_O_2_ ‐induced expression of the pro‐apoptotic factor NOXA contributes to cell death

**Figure 4:**
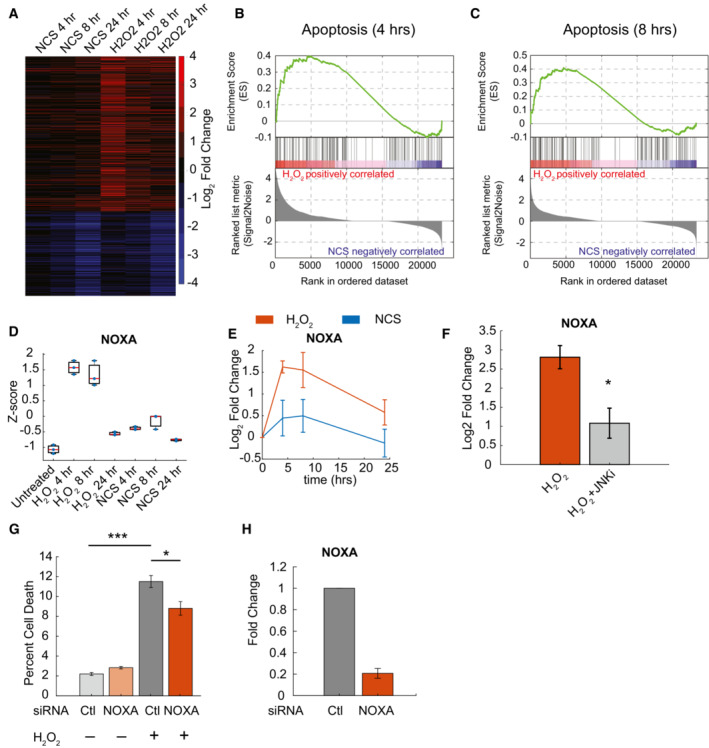
JNK‐dependent expression of NOXA contributes to cell death induction Heatmap of differentially expressed genes (absolute log_2_ fold change > 1, *P*‐value < 0.05) in response to 400 ng/ml NCS or 300 μM H_2_O_2_ over time as compared to untreated control cells. Upregulated genes (red) and downregulated genes (blue) are shown for each condition. Color based on log_2_ fold change of three biological replicates (*n* = 3).GSEA enrichment plot comparing H_2_O_2_‐ and NCS‐treated cells 4‐h post‐treatment.GSEA enrichment plot comparing H_2_O_2_‐ and NCS‐treated cells 8‐h post‐treatment.Box plots showing *Z*‐score distribution for the expression of NOXA for each RNA‐seq condition. Central band represents the median value, with the box bounding the 25^th^–75^th^ percentiles. Whiskers extend to extreme values not considered an outlier (approximately ±2.7σ). Values for three independent biological replicates are shown as blue dots (*n* = 3).Log_2_ fold change for NOXA expression calculated by RT–PCR for NCS‐ and H_2_O_2_‐treated cells over multiple time points. Data represent mean expression ± SEM for three independent biological replicates (*n* = 3).Log_2_ fold change in expression of NOXA in response to 300 μM H_2_O_2_ in the presence or absence of JNK inhibition as compared to untreated control. Samples collected 4‐h post‐treatment at maximum expression identified by RNA‐seq. Data represent mean expression ± SEM based on three biological replicates (*n* = 3). Statistics were calculated using a two‐tailed *t*‐test (*P < 0.05).Percentage of cell death 24‐h post‐H_2_O_2_ treatment in the presence or absence of NOXA knockdown. Data represent mean ± SEM of three independent biological replicates (*n* = 3).Fold change of NOXA expression in response to siRNA knockdown calculated by RT–PCR relative to siRNA control cells. Data represent mean ± SEM of three independent biological replicates (*n* = 3). Data information: *P < 0.05, ***P < 0.001.

ERK, JNK, and p38 each activate transcription factors that control gene expression involved in cell fate determination (Yang *et al*, 2003). Given the difference in cell fate determination between NCS and H_2_O_2_ treatment, we next determined how ERK, JNK, and p38 dynamics cooperate with p53 to differentially regulate downstream gene expression to drive cell fate specification. We performed RNA‐Seq analysis on cells over a range of time points (0, 4, 8, 24 h) to capture dynamic gene expression patterns following treatment with either NCS or H_2_O_2_. We then identified differentially expressed genes for each condition and time point (Fig 4A and Datasets [Link], [Link]). The largest number of differentially regulated genes were induced in response to H_2_O_2_ at 4 h. These findings are consistent with our evidence that MAPKs are activated rapidly in response to H_2_O_2_.

To identify differentially expressed genes that most affected cell death, we performed gene set enrichment analysis (GSEA) (Mootha *et al*, 2003; Subramanian *et al*, 2005) comparing NCS‐ and H_2_O_2_‐treated samples. Given that cell fate determination occurs within the first 8 h (Fig 3G), we focused on gene expression at 4‐ and 8 h following treatment. Significant enrichment of genes associated with apoptotic signaling occurred at both 4 (Fig 4B) and 8 h (Fig 4C) following H_2_O_2_ treatment. Among the highest‐ranked genes was the pro‐apoptotic factor *PMAIP1*, henceforth referred to by its protein name NOXA. *Z*‐scores from comparisons across samples confirmed significant upregulation of both transcripts in H_2_O_2_‐treated cells (Fig 4D), a result validated by RT–PCR (Fig 4E). While NOXA is a well‐characterized p53 target, it has been shown to be regulated by JNK as well (Pietkiewicz *et al*, 2013). Given that JNK dynamics were shown to be important for activating cell death in response to H_2_O_2_ (Fig 3G), we tested whether the upregulation of NOXA was dependent on JNK activity. We inhibited JNK using tanzisertib and measured the changes in expression in response to H_2_O_2_ by RT–PCR (Fig 4F). JNK inhibition significantly reduced the NOXA expression, indicating that its expression depended on JNK activity.

We next determined whether the observed H_2_O_2_‐induced cell death was dependent on the JNK‐dependent increases of NOXA expression. First, we knocked down NOXA expression by siRNA and measured changes in cell death by flow cytometry (Fig 4G). The knockdown reduced the percentage of cell death, albeit not as strongly as JNK inhibition. Knockdown was confirmed by RT–PCR (Fig 4H). Similar modest effects were observed with a second siRNA against NOXA (Appendix Fig S4A and B). These results suggested that H_2_O_2_ drives cell death through JNK‐dependent upregulation of multiple intrinsic apoptosis factors, with NOXA playing a modest role; however, the specific role of p53 dynamics in regulating cell fate in response to H_2_O_2_ treatment remained to be determined.

### p53 regulates the late transcriptional response to H_2_O_2_ and initiates cell cycle arrest in surviving cells

**Figure 5:**
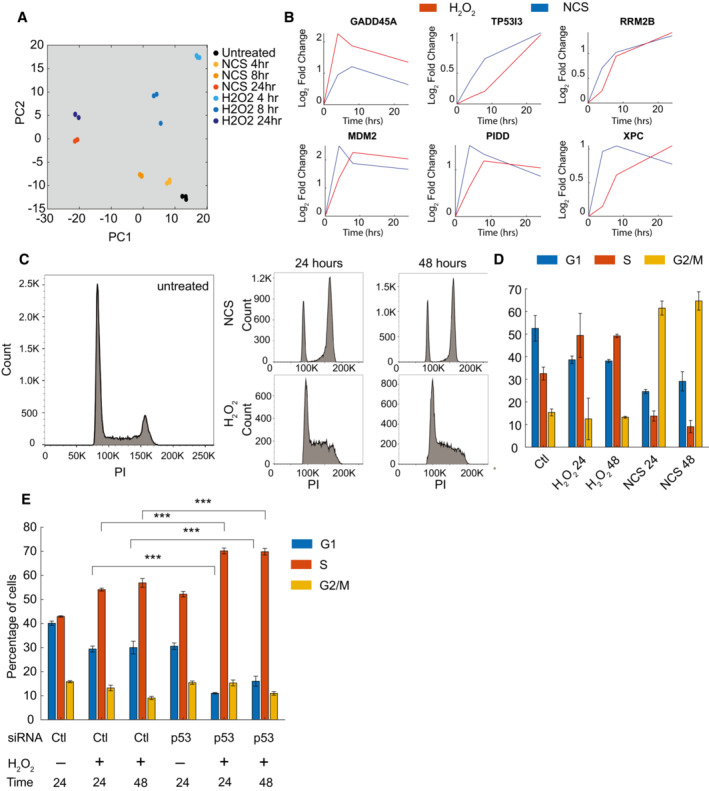
p53 regulates cell cycle progression in response to oxidative stress Principal component analysis (PCA) of RNA‐seq expression data calculated using rLog transformed data from DESeq2 output. Each data point represents a single biological replicate (*n* = 3). Data points are colored according to condition.Plots of select p53 target genes in response to NCS (blue) or H_2_O_2_ (red). Lines represent log_2_ fold change as calculated from RNA‐seq data.Histogram plots illustrating representative cell cycle distributions for control cells and cells treated with either 400 ng/ml NCS or 300 μM H_2_O_2_ over time.Bar graph illustrating the distribution of cells in each cell cycle phase in response to NCS or H_2_O_2_ treatment at both 24‐ and 48‐h post‐treatment. Data represent mean ± SEM of three independent biological replicates (*n* = 3).Bar graph illustrating the distribution of cells in each cell cycle phase in response to H_2_O_2_ treatment with or without knockdown of p53 expression. Data represent mean ± SEM of three independent biological replicates (*n* = 3). Statistics comparing control and knockdown cells performed using one‐way ANOVA (***P < 0.001). Data information: ***P < 0.001.

To identify how p53 dynamics affected cell fate in response to H_2_O_2_, we focused on gene expression changes at the later time points from the RNA‐Seq analysis of the NCS and H_2_O_2_ responses. Principal component analysis (PCA) indicated that gene expression profiles in response to the two stresses became increasingly similar over time (Fig 5A). Furthermore, unsupervised hierarchical clustering grouped the 24‐h time points together (Appendix Fig S5A), and there was an increase in shared differentially expressed genes over time (Appendix Fig S5B). Given that p53 levels were elevated at much later time points compared with the MAPK activities in response to either stress (compare Figs 1C and 2F–H), these results suggested p53 was the dominant factor in regulating later transcriptional responses. Further supporting this hypothesis, while many p53 target genes showed distinct expression patterns in response to the two stresses at early time points, their expression was more similar at later time points as observed via RNA‐seq (Fig 5B).

DNA damage generated by NCS induces cell cycle arrest, and given the similarities in gene expression at later time points between the NCS and the H_2_O_2_ transcriptional responses, we hypothesized that the surviving cells in both conditions preferentially sustained cell cycle arrest. To test this hypothesis, we performed cell cycle analysis to compare the cell cycle distribution of 400 ng/ml NCS‐ and 300 μM H_2_O_2_‐treated cells over time (Fig 5C). Sub‐G1 cells were excluded from the analysis to specifically focus on changes in cell cycle distribution in cells that survived treatment. We observed that NCS treatment led to a strong accumulation of cells in G2/M. Cells treated with 300 μM H_2_O_2_ underwent cell cycle arrest, though with a greater percentage of cells progressing into S‐phase compared with the response to NCS treatment (Fig 5D). To determine the contribution of p53 in maintaining cell cycle arrest, we knocked down p53 expression by siRNA prior to H_2_O_2_ treatment. We observed that p53 knockdown led to an increased transition from G1 to S, as the percentage of cells in G1 decreased and the percentage in S‐phase increased (Fig 5E), suggesting that p53 was the dominant factor in regulating the G1‐to‐S transition in surviving cells in response to H_2_O_2_.

### p38 cross‐talk balances cell fate determination by regulating p53 expression and JNK activity

**Figure 6:**
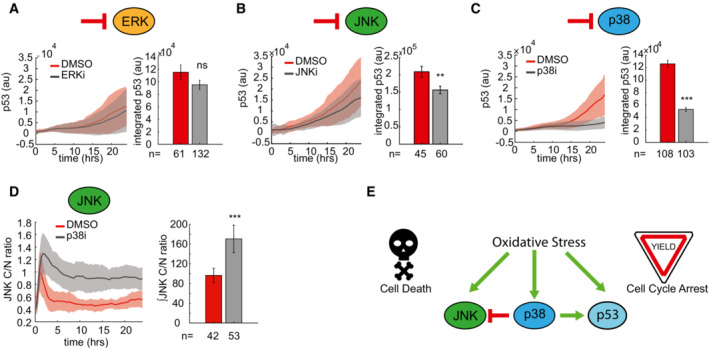
p38 activity regulates both JNK and p53 activity A–C
Measuring mean p53‐fluorescent p53 levels in individual cells in response to 300 μM H_2_O_2_ with or without ERKi (A), JNKi (B), or p38i (C). Control cells are shown in red and inhibitor‐treated cells in gray. Bar graphs show integrated levels of fluorescent p53 over the 24‐h time course. Error bars ± SEM. Statistics were performed using a 2‐tailed *t*‐test. Number of analyzed cells, *n*, is shown for each condition.D
Mean JNK activity as measured by C/N ratio (bold line) ± SD (shaded area) in response to 300 μM H_2_O_2_ with or without p38 inhibition (gray). Bar graph shows integrated JNK activity (C/N ratio) over 24‐h. Data represent mean ± SD. The number of analyzed cells, *n*, is shown for each condition. Statistics were performed using a 2‐tailed *t*‐test.E
Schematic highlighting the cross‐talk between JNK, p38, and p53 in response to oxidative stress and the observed roles in cell fate determination. Data information: **P < 0.01, ***P < 0.001, ns = not significant.

Thus far, our data supported independent roles for the MAPKs and p53 in response to oxidative stress, with JNK activity inducing cell death and p53 expression regulating cell cycle progression. However, several potential mechanisms for cross‐talk between the MAPKs and p53 have been identified in a variety of conditions (Fuchs *et al*, 1998; She *et al*, 2000; Malmlöf *et al*, 2007). To identify possible cross‐talk between the MAPKs and p53 in the response to H_2_O_2_, we first focused on the ability of the MAPKs to potentially regulate p53 expression dynamics by altering p53 stability as one potential mechanism of cross‐talk. To test whether MAPK‐dependent p53 stabilization occurred in response to H_2_O_2_, we selectively inhibited ERK (Fig 6A), JNK (Fig 6B), or p38 (Fig 6C) and measured the impact on H_2_O_2_‐induced p53 accumulation in individual cells over 24‐h. p38 inhibition led to a significant reduction in the accumulation of p53 over time, whereas ERK and JNK inhibition had modest effects. This finding suggests that p38 activation is important for the regulation of cell cycle arrest by regulating the accumulation of p53.

Next, we determined whether cross‐talk between MAPKs contributed to the cellular response to H_2_O_2_. Inhibition of p38 increased the percentage of cell death (Fig 3B), which, given the importance of JNK activity in inducing cell death in response to H_2_O_2_ (Fig 3G), suggested that p38 activity antagonizes JNK activity. Previous studies identified similar cross‐regulation between p38 and JNK in the regulation of UV‐induced cell death (Miura *et al*, 2018). To test whether such cross‐talk also exists in response to H_2_O_2_ treatment, we inhibited p38 using FHPI and measured JNK activity in single cells in response to H_2_O_2_. We found that p38 inhibition prolonged the activation of JNK (Fig 6D). Taken together, these results suggest that p38 alters the balance of cell fate determination in response to H_2_O_2_ treatment through cross‐talk with both p53 and JNK. Specifically, p38 activity may enhance p53‐dependent cell cycle arrest by upregulating p53 expression and attenuate JNK‐dependent cell death through inhibition of JNK activity (Fig 6E).

## Discussion

DNA is constantly damaged through biological processes and environmental agents with estimates of tens of thousands of lesions per day per cell (Lindahl & Barnes, 2000; Jackson & Bartek, 2009). To maintain genomic integrity these lesions must be resolved. Despite the repair of specific DNA lesions typically requiring unique molecular machinery (Lindahl *et al*, 1982; Sargent *et al*, 1997; Sugasawa *et al*, 2001), the tumor suppressor p53 is a key regulator of cell fate in response to a variety of cellular stresses (Horn & Vousden, 2007). These observations raise the question of how cells distinguish between different cellular stresses to enact distinct cell fate decisions and transcriptional responses. Single‐cell studies have provided insight into this question, as p53 dynamics can vary across stresses (Batchelor *et al*, 2011), diversify gene expression patterns (Porter *et al*, 2016; Hafner *et al*, 2017), and regulate specific cell fates (Purvis *et al*, 2012; Paek *et al*, 2016; Yang *et al*, 2018). However, studies are increasingly finding p53 dynamics to be complex; existing across a spectrum (Stewart‐Ornstein & Lahav, 2017) and switching behavior over time and DNA damage dose (Yang *et al*, 2018; Tsabar *et al*, 2020). We found that the information conveyed by p53 dynamics is limited in that different stresses can induce quantitatively similar p53 expression patterns. However, we also observed that cells can still induce distinct cell fate responses independent of p53 dynamics, suggesting that cells make use of additional mechanisms in conjunction with p53 dynamics to dictate cell fate.

One mechanism to improve the specificity of response is to incorporate multiple signaling networks, and increasingly systems biology approaches are revealing how cells make use of multiple networks to integrate and process biological signals (Antebi *et al*, 2017; Miura *et al*, 2018; De *et al*, 2020). Here, we showed how the integration of stress‐responsive MAPK activation in conjunction with p53 can increase specificity in the cellular responses to DNA double‐strand breaks and oxidative stress. We found that ERK, JNK, and p38 are preferentially activated in response to H_2_O_2_‐induced oxidative stress but not DNA double‐strand breaks induced by NCS. This is in contrast to work in MCF7 cells, which identified pulses of ERK activation in response to NCS (De *et al*, 2020). These differences may reflect differences in growth conditions, as hTERT‐HPNE require exogenous EGF resulting in high basal ERK signaling. Similarly, co‐treatment of NCS and heregulin led to a sustained ERK response and no obvious pulses (De *et al*, 2020). Unlike p53, we found that the kinases contributed to the induction of cell death, with ERK and JNK activation promoting cell death and p38 activation inhibiting it. While Annexin V and propidium iodide staining is typically indicative of apoptosis, other mechanisms of cell death are possible and further study is needed to elucidate the precise mechanism of cell death. Results from previous studies of JNK suggested that JNK activation dynamics contribute to cell fate regulation, with transient activation favoring survival (Sluss *et al*, 1994; Chen *et al*, 1996a; Lee *et al*, 1997) and sustained activation favoring cell death (Chen *et al*, 1996b; Guo *et al*, 1998). In response to endoplasmic reticulum (ER) stress, a two‐phase role for JNK has been proposed where early JNK activation is anti‐apoptotic while late JNK activation favors cell death (Brown *et al*, 2016). To determine whether the dynamics of the activity of JNK or the other MAPKs similarly affect the response to oxidative stress, we used KTR reporters to quantify MAPK activities in real time in single living cells. We found that, indeed, early, repeated activation of JNK was associated with cell death in response to H_2_O_2_‐induced stress. Furthermore, the timing of JNK re‐activation occurs at ~6 h after stress induction, similar to the timing of the second phase of JNK activation observed in response to ER stress. How these dynamics impact the transcriptional response of surviving and dying cells is an important question, and future studies isolating the two groups should provide insight into how the transcriptional responses diverge to generate stress‐specific cell fate responses.

From RNA‐Seq analysis, we identified a number of pro‐apoptotic factors (Lomonosova & Chinnadurai, 2008), that were strongly differentially regulated between conditions of oxidative stress and DNA damage at early time points. Expression of NOXA in particular was strongly regulated by JNK activation. Previously, it was demonstrated that the JNK1 isoform preferentially induced the expression of NOXA in response to proteasome inhibition (Pietkiewicz *et al*, 2013). A drawback of the current JNK KTR is the inability to distinguish between the JNK1 and JNK2 isoforms; however, in our western blot validation of JNK activation, we observed that H_2_O_2_ treatment primarily led to phosphorylation of the p46 JNK1 isoform, supporting JNK1‐specific induction of NOXA. Given the evidence of isoform‐specific function, further studies of differential dynamics between isoforms will be crucial to understanding how JNK dynamics contribute to the cellular stress response. We also found that the knockdown of NOXA levels was able to partially attenuate cell death in response to H_2_O_2_ treatment, though not fully. The partial reduction may be due to compensatory upregulation of alternative apoptotic factors, and a similar mechanism was recently observed in T cells (Ludwig *et al*, 2020). Further study is needed to determine how dynamic regulation of pro‐ and anti‐apoptotic factors contribute to cell death in response to genotoxic stresses.

While the majority of differentially expressed genes induced in response to H_2_O_2_ likely reflect differential activation of MAPK‐regulated transcription factors such as c‐Jun, we also observed altered patterns in the expression of specific p53 target genes despite similar p53 dynamics between stresses. The differential regulation is potentially due to differences in co‐factors that coordinate with p53 to regulate specific target genes, and MAPKs have been proposed to affect the expression of several p53 target genes including *MDM2* (Ries *et al*, 2000), *GADD45A* (Daino *et al*, 2006), and *BBC3* (Cazanave *et al*, 2009). An alternative possibility is that the differential regulation of p53 targets is dependent on differences in p53 phosphorylation status in response to the different stresses, and MAPKs have been shown to post‐translationally modify p53 (Fuchs *et al*, 1998; She *et al*, 2000). Furthermore, specific phosphorylation sites on p53 have been proposed to control the expression of specific target genes (Oda *et al*, 2000); however, in previous studies, all post‐translational modified forms of p53 exhibited dynamics similar to that of total p53 expression (Loewer *et al*, 2010). Further study of these mechanisms will be necessary to determine how they contribute to the fine‐tuning of the expression of specific target genes and the diversification of stress responses.

Determining how p53 dynamics control cell fate is an active area of investigation, and several mechanisms have been identified in different stress response contexts. p53 dynamics have been shown to enable several higher‐level signal processing functions: pulse counting, where a certain number of pulses promotes cell death (Sun *et al*, 2009; Zhang *et al*, 2009); integration, in which integrated rather than threshold concentrations of p53 determine the trigger for cell death (Paek *et al*, 2016); and switching, in which a qualitative change in p53 dynamics changes the cell state from pro‐survival to pro‐death (Yang *et al*, 2018). In response to oxidative stress, we found that p53 is important for regulating longer‐term cell cycle arrest, and that the dynamics of JNK is the key factor in activating cell death in the early response. These findings suggest a mechanism whereby cells can integrate multiple pathways with distinct temporal dynamics to govern cell fate decisions across temporal scales ‐‐ an early cell death response with a later cell cycle arrest. We showed that p38 activity balances the two responses by hindering JNK activation and promoting p53 expression. Manipulating the balance between p53‐dependent and independent mechanisms may have value in cancer treatments where p53 is typically mutated, i.e., if tumor cells can be preferentially shunted to a p53‐independent response they may be more responsive to therapy. Further study of the role of p53 during other p53‐independent cell death responses is necessary to determine whether this mode of regulation is specific to oxidative stress or more generalizable across stresses.

## Materials and Methods

### Plasmids

Plasmid for expression of fluorescently tagged H2B, pLentiPGK Hygro DEST H2B‐mCerulean3, was obtained from Addgene (Plasmid #90234). Plasmids for ERK (pLentiCMV Puro DEST ERKKTRClover), JNK (pLentiPGK Puro DEST JNKKTRClover), and p38 (pLentiPGK Puro DEST p38KTRClover) kinase translocation reporters were obtained from Sergi Regot; however, these plasmids are also publicly available via Addgene and catalog numbers are listed in Key Resource Table. Plasmid for the expression of mCherry tagged p53, pRRLBsd‐pMT‐p53‐mCherry was generated by gateway cloning of the p53 cDNA sequence.

Packaging of plasmids into lentivirus was performed using the Takara Lenti‐X Packaging Single shot kit (631275) and 293T cells (ATCC, CRL‐11268) according to manufacturer specifications. Virus‐containing media were collected at 48‐ and 72‐h postinfection, filtered, and concentrated 10‐fold using Takara Lenti‐X concentrator (631231). Virus production was confirmed by Lenti‐X GoStix (Takara, 631280). Lentivirus was stored in aliquots at −80°C until use.

### Human cell lines and culture

The immortalized nontransformed pancreatic cell line hTERT‐HPNE was obtained from the ATCC (CRL‐4023) and was maintained in growth media consisting of 75% DMEM without glucose (ThermoFisher, 11966025) and 25% M3 BaseF media (InCell, M300F). Media were supplemented with 5% FBS, 1× Amphotericin B/penicillin/streptomycin mix (Fisher Scientific, 50‐751‐7247), 5.5 mM D‐glucose (VWR, MK491212), and 750 ng/ml puromycin. 10 ng/ml EGF (Peprotech, AF‐100‐15) was added fresh to media prior to use with cells. Henceforth referenced as HPNE media.

To establish fluorescent HPNE cell lines, cells were transduced with lentivirus‐containing pLentiPGK Hygro DEST H2B‐mCerulean3 (Addgene: 90234) diluted 1:50 in HPNE media supplemented with 8 μg/ml protamine sulfate and 10 μM HEPES. After 72 h, media were removed and selection began in HPNE media supplemented with 500 μg/ml hygromycin. Selected cells were then transduced with lentivirus containing one of either pLentiCMV Puro DEST ERKKTRClover, pLentiPGK Puro DEST JNKKTRClover, or pLentiPGK Puro DEST p38KTRClover. Positive cells were isolated via FACS. Lastly, fluorescent p53 was introduced by transducing cells with lentivirus‐containing pRRLBsd‐pMT‐p53‐mCherry. Positive cells were selected using 5 μg/ml blasticidin and single‐cell clones were established by limiting dilution. Expression of H2B‐mCerulean, KTR Clover, and p53‐mCherry was confirmed by fluorescence microscopy. All cells were maintained at 37°C and 5% CO_2_.

Cell lines were routinely tested for mycoplasma contamination (Millipore‐Sigma).

### Live‐cell microscopy of p53 and kinase dynamics

For live‐cell imaging, cells were plated into glass‐bottom dishes (Mattek) and were imaged 24 h after plating. Two hours prior to imaging, the medium was replaced with transparent DMEM medium lacking riboflavin and phenol red (ThermoFisher, custom formulation) and supplemented with 2% fetal bovine serum (FBS) and 1× Amphotericin B/penicillin/streptomycin mix (Fisher Scientific, 50‐751‐7247). For conditions involving MAPK inhibition, inhibitors were added at this stage with untreated cells receiving a DMSO control dose. After 2 h, cells were imaged with a Nikon Eclipse Ti‐inverted fluorescence microscope equipped with an automated stage (Prior) and a custom chamber to maintain the constant 37°C temperature, high humidity, and 5% CO_2_. Stage positions were set to capture multiple wells and positions per experiment. The first frame was imaged to capture the untreated baseline activity and then NCS or H_2_O_2_ was added between the first and second frames. Images were acquired every 8 min for 24 h using a YFP filter set (Chroma) (488–512 nm excitation filter, 520 nm dichroic beam splitter, and 532–554 nm emission filter) to capture kinase translocation reporters, a TRITC filter set (Chroma) (540–580 nm excitation filter, 585 nm dichroic beam splitter, and 593–668 nm emission filter) to capture p53 dynamics, and a CFP filter set (Chroma) (426–446 nm excitation filter, 445 nm longpass dichroic beam splitter, 460–500 nm emission filter) to capture the H2B nuclear marker. Images were collected using a 20× CFI Plan Apochromat Lambda (NA = 0.75) objective (Nikon). For each condition, we attempted to measure a minimum of 40 individual cells. Following imaging, data were exported as individual tiffs for each channel, position, and time point.

### Quantification of nuclear p53‐mCherry and kinase C/N ratio

Using the exported H2B‐mCerulean images, nuclei were segmented using CellProfiler software (v.3.1.9) to generate a grayscale mask identifying the nuclei of all cells using the IdentifyPrimaryObjects function. Cells were automatically tracked using the Cell Profiler TrackObjects function using the overlap method with a 50‐pixel maximum distance to consider a match. Tracked cells were confirmed by manual visualization of image stacks. Nuclear masks were used to define regions of interest and mean fluorescence intensity for p53‐mVenus images was determined in an automated manner using custom Matlab scripts for these regions. In instances where automatic tracking failed to provide sufficient cell numbers, regions of interest were defined by manually defining nuclei locations using polygons. For analysis of cells that underwent cell death, nuclei were defined manually and dying cells identified by visual morphology. Cells that underwent cell death rounded up and lost fluorescence at the time of death.

Once nuclei were defined mean fluorescence intensity for p53‐mCherry was calculated for these regions using the same Matlab scripts. To calculate cytoplasmic‐to‐nuclear ratios for the kinase translocation reporters nuclear masks were grown by 5 pixels and the nuclear mask was subtracted to generate a cytoplasmic ring as previously described (Regot *et al*, 2014). Mean fluorescence intensity of the KTR‐Clover channel was measured for both the nuclear region and cytoplasmic ring. C/N ratio was calculated by dividing the resulting mean fluorescence values of each compartment. For plotting, values were smoothed using a moving mean average with a three‐window span (*n* − 1, *n*, *n* + 1). *n* represents the number of individual cells analyzed for each plot and is discussed in figure legends.

### Quantification of dynamic features of kinase activation

Quantification of pulse number, maximum amplitude, peak timing, and peak duration was performed using custom Matlab software. Using the calculated C/N ratio for each kinase, pulses of activity were identified using the findpeaks function in the Matlab Signal Processing toolbox to identify local maxima within single‐cell traces. To minimize spurious identification of peaks due to noise in expression, we defined a minimum peak prominence as 50% of the difference between the median basal and peak expression of all cells analyzed for that condition. Using these identified maxima, we could further quantify amplitude, timing, and duration based on the timing of these maxima. In the case of p38, which largely exhibited a single pulse of activity we specifically focused on the maximum C/N ratio and the timing of the maximum C/N ratio. Data for pulse number represent the number of kinase pulse numbers observed over 24‐h. All data are presented using boxplots to show the spread of population data. Outliers are shown as individual blue circles. All statistics were performed using one‐way ANOVA for multiple comparisons with a *P*‐value < 0.05 determined as significant. *n* represents the number of individual cells analyzed and is reflected in the figures and figure legends.

### Western blot analysis

Cells were cultured in either 35‐mm or 6‐cm dishes for western blot analysis. Culture medium was removed from cells by aspiration, and cells were washed once with 1× PBS. Cells were then scraped into 1 ml of 1× PBS, and the plate was rinsed with an additional 1 ml of PBS added to the collected cells. Cells were pelleted by centrifugation for 10 min at 13,000 rpm and flash‐frozen in a dry ice:EtOH bath. Cell pellets were lysed in lysis buffer (50 mM Tris–pH 7.5, 100 mM NaCl, 0.5% sodium deoxycholate, 1% Triton‐X, 0.1% SDS) supplemented with 50 mM NaF, 1 mM PMSF, and 1:100 phosphatase inhibitor I (Sigma, P2850). Lysates were incubated on ice for 30 min, and insoluble debris was pelleted by centrifugation. Protein concentration was determined by Bradford assay (Biorad, 5000006). 20 μg of total lysate were run per lane and separated using 4–20% Tris–Glycine gels (Biorad, 5671095). Proteins were transferred to Immobilon‐FL PVDF membrane (Millipore, IPFL00010). Membrane sections were blocked for 1 h in Licor Blocking Buffer (Licor, 927‐4000) and incubated with primary antibodies overnight. Membranes were washed 3× with 0.1% PBST before incubation with IrDye secondary antibodies (1:5,000) for 1 h. Membranes were washed three times with PBST and imaged using an Odyssey Imager.

All primary antibodies were used at a concentration of 1 μg/ml unless otherwise indicated. The following antibodies were used: mouse monoclonal anti‐p53 DO‐1 (Santa Cruz Biotechnology, sc‐126) [1:1,000], rabbit monoclonal anti‐β‐Actin (Cell Signaling Technology, 4970) [1:3,000], mouse monoclonal anti‐ERK1/2 (Cell Signaling Technology, 4696) [1:1,000], rabbit monoclonal anti‐phospho‐ERK1/2 (Thr202/Tyr204) (Cell Signaling Technology, 4370) [1:1,000], rabbit polyclonal anti‐SAPK/JNK (Cell Signaling Technology, 9252) [1:1,000], rabbit polyclonal anti‐phospho‐SAPK/JNK (Thr183/Tyr185) (Cell Signaling Technology, 9251) [1:1,000], rabbit polyclonal anti‐p38 (Cell Signaling Technology, 9212) [1:1,000], rabbit monoclonal anti‐phospho‐p38 (Thr180/Tyr182) (Cell Signaling Technology, 4511) [1:1,000]. Secondary antibodies used were anti‐Mouse 680 RD and anti‐Rabbit 800 CW (Licor) and were used at a dilution of 1:5,000. Experiments were performed using three biological replicates.

### siRNA knockdown

1.5 × 10^5^ hTERT‐HPNE cells were plated into 6‐cm plates (Genesee) and allowed to adhere for 4‐h. siRNA was prepared according to manufacturer specifications (Horizon) and all siRNA used within this study are described in the Key Resources Table. Briefly, siRNA was diluted to 5 μM in RNase‐free water. For each reaction 4 μl of siRNA was mixed with 196 μl Optimem‐I (ThermoFisher, 31985070) to prepare tube A. Tube B contained 4 μl of Dharmafect‐I and 196 μl Optimem‐I. After 5 min, tubes A and B were mixed and allowed to incubate for 20 min at room temperature. Media were removed from cells, and 400 μl of siRNA mixture was added along with 1.6 ml growth media for a final volume of 2 ml with a final siRNA concentration of 10 nM. Unless otherwise specified all siRNA knockdowns were performed at 10 nM concentration. After 18‐h transfection, media were removed and replaced with fresh media. One more media exchange occurred at 48‐h post‐transfection. Functional assays (cell death, cell cycle) were performed 48‐h post‐transfection. siRNA knockdowns were validated by either western blot analysis or RT–PCR.

### qRT–PCR

3.5 × 10^5^ hTERT‐HPNE cells were plated in 6‐cm plates (Genesee) and allowed to grow for 24 h. For comparison of NCS and H_2_O_2_‐induced NOXA cells were treated with either 400 ng/ml NCS or 300 μM H_2_O_2_. mRNA was isolated using the Qiagen RNeasy plus kit (Qiagen, 74134) according to manufacture specifications at 0‐, 4‐, 8‐, and 24‐h post‐treatment. For analysis of JNK‐dependent expression of NOXA cells were pretreated for 2 h with tanzisertib or equal volume DMSO control prior to the addition of 300 μM H_2_O_2_. mRNA was isolated using the Qiagen RNeasy plus kit (Qiagen, 74134) according to manufacture specifications at 0‐ and 4‐h post‐treatment. RNA concentration was determined by Nanodrop (ThermoFisher), and cDNA was prepared using the High‐Capacity cDNA Reverse Transcription kit in 20 μl reactions (ThermoFisher, 4374966) according to manufacturer specifications. After cDNA generation, the final volume was brought to 100 μl. RT–PCR was performed in 10 μl reactions containing 5 μl Maxima SYBR green master mix (ThermoFisher, K0222), 2 μl H_2_O, 2 μl cDNA, and 1 μl of 10 μM specific primers. Primers used include *GAPDH* Forward 5′‐ACATCGCTCAGACACCATG‐3′, *GAPDH* Reverse 5′‐TGTAGTTGAGGTCAATGAAGGG‐3′, NOXA Forward 5′‐GCAAGAACGCTCAACCGAG‐3′, NOXA Reverse 5′‐TCCTGAGCAGAAGAGTTTGGAT‐3′, Log_2_ Fold Change was calculated by ΔΔCt using *GAPDH* as a loading control. All experiments were performed using three biological replicates.

For quantification, we normalized the expression of each p53 target to its on‐plate *GAPDH* expression control and quantified fold change by ΔΔCt, comparing each time point to the expression at time zero. *n*, discussed in figure legends indicates the number of independent biological replicates for each condition and time point. Data presented as mean log_2_ fold change ± SEM. Statistics were performed using unpaired two‐tailed *t*‐tests to compare control and experimental groups. Significance was defined as *P* < 0.05.

### Cell cycle analysis

3.5 × 10^5^ hTERT‐HPNE cells were plated onto 6‐cm plates (Genesee) and allowed to grow for 24 h. For siRNA‐treated cells, 1.5 × 10^5^ hTERT‐HPNE cells was plated onto 6‐cm plates and transfected as described. At the time of treatment, media were replaced with fresh media and treated cells received either 400 ng/ml NCS or 300 μM H_2_O_2_. After 24 or 48 h, media were collected and placed into labeled tubes. Cells were trypsinized and added to the collection tube containing the original media. Cells were spun down at 400 *g* for 4 min and washed twice with PBS to remove residual media. Cell pellets were re‐suspended in 300 μl PBS and passed through a 45 μm cell strainer to remove clumps. Cells were then fixed by the addition of 700 μl cold ethanol dropwise. Fixed cells were stored at −20°C until staining. Prior to staining, cells were spun down at 750 *g* for 5 min and washed twice with PBS to remove ethanol. 1 × 10^6^ cells were then re‐suspended in 250 μl PBS + 10 μl propidium iodide and 5 μl RNaseA. Cells were incubated at 37°C for 30 min, then brought to 500 μl final volume by PBS and passed through a 45 μm cell strainer prior to analysis on a BD LSR II from the University of Minnesota Flow Cytometry Core. Single cells were identified from the BD LSR II data based on initial gating on forward and side scatter measurements followed by PI‐A and PI‐W gating. Sub‐G1 cells were excluded from the analysis to exclusively focus on changes to cell cycle distribution among cells that survive exposure to stress. A minimum of 10,000 cells were collected per condition, except for siRNA knockdown of p53 studies where fewer cells were observed and an average of 5,000 cells were analyzed per condition. Cell cycle distributions for cells in G1, S, or G2/M phases were performed using FlowJo v.10 and the Watson Pragmatic algorithm. Data for cell cycle distributions were presented as the mean ± SEM for a minimum of three biological replicates. Statistical analysis was performed by one‐way ANOVA for multiple comparisons. Significance was defined as *P* < 0.05. *n*, discussed in figure legends represents the number of independent biological replicates performed for each experiment.

### Cell death analysis

3.5 × 10^5^ hTERT‐HPNE cells were plated onto 6‐cm plates (Genesee) and allowed to grow for 24 h. Media were replaced with fresh media and treated cells received either 400 ng/ml NCS or 300 μM H_2_O_2_. For cells treated with specific inhibitors, cells were treated for 2 h prior to the addition of H_2_O_2_ unless otherwise indicated. Control cells for inhibitor‐treated groups received an equal volume of DMSO treatment. 24‐h post‐treatment, media were collected and placed into labeled tubes. Cells were then trypsinized and added to the collection tube containing the original media. Cells were spun down at 400 *g* for 4 min and washed twice with PBS to remove residual media. After washes cells were labeled using AnnexinV‐APC and propidium iodide (PI) according to manufacturer specifications (Biolegend, Cat #640932). Cells were then analyzed using a BD LSR II through the University of Minnesota Flow Cytometry Core.

All cells were identified from the BD LSR II data based on initial gating on forward and side scatter measurements. Compensation controls using single staining of cells with AnnexinV‐APC or prodium iodide were used to establish gating of APC‐ and PI‐positive cells. AnnexinV‐APC and PI staining was plotted as a biplot with four quadrants (double negative, APC positive, PI positive, and double positive). Cell death was quantified for each sample as the percentage of double‐positive (AnnexinV‐APC and PI positive) cells in each sample. For each biological replicate a minimum of 30,000 cells were analyzed. Data presented represent mean percentage ± SEM of cell death for a minimum of three biological replicates. *N*, discussed in figure legends represents the number of independent biological replicates performed for each experiment. Statistical analysis was performed by one‐way ANOVA for multiple comparisons. Significance was defined as *P* < 0.05.

### RNA‐seq

3.5 × 10^5^ hTERT‐HPNE cells were plated in 6‐cm plates (Genesee) and allowed to grow for 24 h. Media were replaced with fresh media and cells were treated with either 400 ng/ml NCS or 300 μM H_2_O_2_. RNA was isolated at 0‐, 4‐, 8‐, and 24‐h post‐treatment using the RNeasy Plus Minikit (Qiagen, Cat #74134) according to manufacturer specifications. For each time point and condition, we collected three biological replicates. Following isolation, RNA concentration was determined by Nanodrop, and RNA was submitted to the University of Minnesota Genomics Core for sequencing. RNA integrity was confirmed by Agilent TapeStation and library prepared using TruSeq Stranded mRNA library prep. After library preparation, samples were sequenced using 150 paired‐end reads on the Novaseq 6000 system (Illumina).

All RNA‐seq analysis was performed using a custom Galaxy server through the University of Minnesota Supercomputing Institute. Analysis was based on three biological replicates per condition (*n* = 3). For analysis of differentially expressed genes, we aligned reads to the genome using Hisat2 with default settings. Aligned reads were counted using the htseq‐count module within Galaxy. Counted reads were then input into DESeq2 to identify differentially expressed genes. To enable comparisons between both untreated and treated samples, as well as across treatments, we used the output of all levels versus all levels. From the calculated log_2_ fold changes, we defined differentially expressed genes as those exhibiting an absolute log_2_ fold change > 1 and an adjusted *P*‐value < 0.05. Heatmap plotting of differentially expressed genes was performed using Matlab 2020a. Genes were clustered based on two general clusters (up‐ and downregulated) identified using the silhouette function and then plotted using the heatmap function for Fig 4. For hierarchical clustering of differentially expressed genes used in Appendix Fig S4 the clustergram function was used.

Additional output from DESeq2 included rLog normalized counts. *Z*‐score analysis of NOXA was performed using rLog normalized count data for each sample. *Z*‐score was calculated for each sample according to the formula.

$$Z=\frac{x‐\mu }{\sigma }$$

where *x* is the observed rLog normalized count for a given gene in a given sample, *μ* is the mean rLog count for the same gene across all samples, and *σ* is the standard deviation of the rLog counts for the gene across samples. *Z*‐scores were plotted using box plots with each individual data point shown as a blue circle. Data were based on three biological replicates (*n* = 3), discussed in figure legends.

Principal component analysis (PCA) in Fig 5 was also performed using rLog normalized values output from DESeq2. Principal component scores were calculated for each sample using the pca function in Matlab 2020a and plotted for all samples. Each data point is represented for a specific sample by a colored dot (*n* = 3) for three biological replicates. *N*, discussed in figure legends.

### Gene set enrichment analysis (GSEA)

Gene set enrichment analysis was performed using the GSEA program (Mootha *et al*, 2003; Subramanian *et al*, 2005). Normalized counts from DESeq2 were used for the expression data. For our analysis, we focused on genes that were enriched between NCS‐ and H_2_O_2_‐treated samples and thus examined the 4‐ and 8‐h time points of these two responses. For plotting graphs, the max number of plots was adjusted upward to 25.

### Materials availability

Plasmid for H2B‐mCerulean3 fluorescent nuclear marker is publicly available from the Markus Covert lab (Kudo *et al*, 2018) on Addgene (pLentiPGK Hygro DEST H2B‐mCerulean3, Plasmid #90234). Plasmids for the ERK, JNK, and p38 kinase translocation reporters were obtained from Sergi Regot (Regot *et al*, 2014), however, are also publicly available through Addgene (pLentiCMV Puro DEST ERKKTRClover, Plasmid #59150; pLentiPGK Puro DEST JNKKTRClover, Plasmid #59151; pLentiPGK Puro DEST p38KTRClover, Plasmid #59152) Plasmid for the expression of p53‐mCherry is available upon request through the Lead Contact, Eric Batchelor.

## Author contributions

**Ryan L Hanson:** Conceptualization; resources; data curation; software; formal analysis; validation; investigation; visualization; methodology; writing – original draft; writing – review and editing. **Eric Batchelor:** Conceptualization; resources; formal analysis; supervision; funding acquisition; investigation; visualization; methodology; writing – original draft; writing – review and editing.

## Disclosure and competing interests statement

The authors declare that they have no conflict of interest.

## Supporting information



Appendix

Dataset EV1

Dataset EV2

Dataset EV3

Dataset EV4

Dataset EV5

Dataset EV6

## Data Availability

Single‐cell KTR and p53 expression data for all single‐cell studies within the manuscript have been deposited in Github (https://github.com/BatchelorLab/Hanson_and_Batchelor_MSB_p53_MAPK) and are available for download as a Matlab data file. Each file contains a cell array containing either single‐cell kinase data or p53 expression data. A cell array with the names for each condition and a data matrix containing each time point are included within each data file to facilitate easy reproduction of the plotted data presented within this manuscript. All analysis code was generated using Matlab R2020a, and specific code to reproduce plotted data is available in Github. All RNA‐seq data are publicly available through GEO (GSE188458).
