# Supplementary material for: Coordination of MAPK and p53 dynamics in the cellular responses to DNA damage and oxidative stress
Source: Mol Syst Biol. 2022 Dec 6;18(12):e11401. doi: 10.15252/msb.202211401 (PMC9724178; doi:10.15252/msb.202211401)
Supplement: Supplementary file 1 — Appendix [file MSB-18-e11401-s004.pdf]

Appendix For

Coordination of MAPK and p53 dynamics in the cellular responses to DNA damage and oxidative stress

Ryan L. Hanson[1] and Eric Batchelor[1,2,*]

Table of Contents

- Appendix Figure S1: H_2O_2-induced cell death is independent of p53
- Appendix Figure S2: MAPKs are activated in response to high dose H_2O_2-
- Appendix Figure S3: Impact of ERK, JNK, and p38 on H_2O_2-induced cell death
- Appendix Figure S4: Knockdown of NOXA modestly reduces H_2O_2-induced cell death
- Appendix Figure S5: Late gene expression patterns of NCS and H_2O_2 are similar

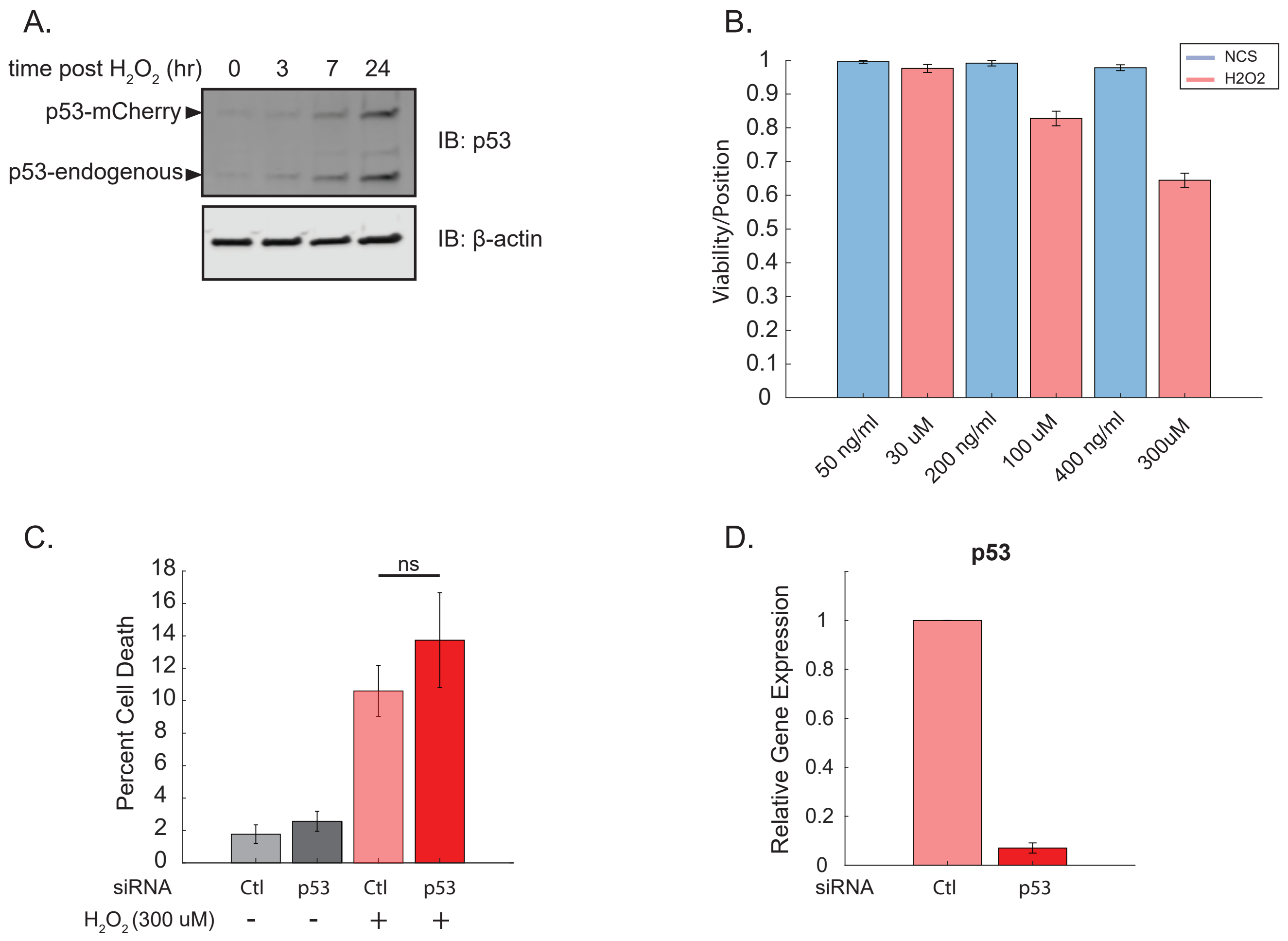

Appendix Figure S1. H_2O_2-induced cell death is independent of p53, related to Figure 1.
A) Western blot assessing expression dynamics of p53-mCherry and endogenous p53 in response to 300 μM H_2O_2 at indicated time points. Image is representative of three biological replicates B) Cell viability as assessed by morphology in live cell imaging experiments. Data represents the mean±SEM of seven imaging fields (n=7) C) Percentage of cell death at 24-hours in response to 300 μM H_2O_2 with or without p53 knockdown. ns=not significant. Data represents the mean±SEM of three biological replicates (n=3) D) Confirmation of p53 knockdown by siRNA by RT-PCR. Data shown as Fold Change relative to control cells receiving non-targeting control. Data represents the mean±SEM of three biological replicates (n=3).

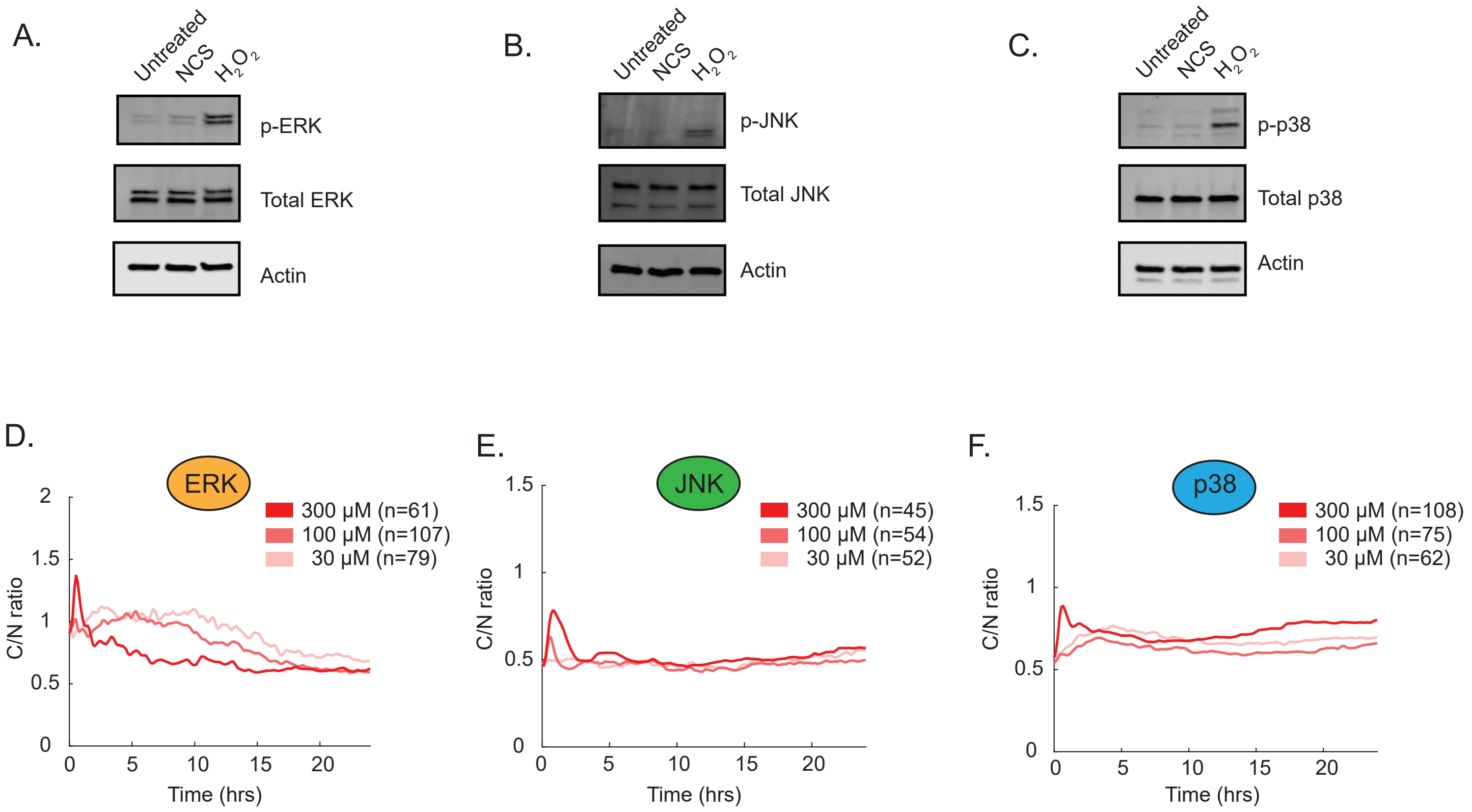

Appendix Figure S2. MAPKs are activated in response to high dose H_2O_2, related to Figure 2.
A-C) Western blot analysis of phosphorylated ERK (A), JNK (B), or p38 (C) in response to 30 minutes NCS or H_2O_2 treatment. Blots are a representative sample from 3 biological replicates. D-F) Average kinase activity as measured by C/N ratio of kinase biosensors for ERK (D), JNK (E), or p38 (F) in response to a dose range of H_2O_2. Number of cells (n) analyzed per condition is shown within the figure next to each dose tested.

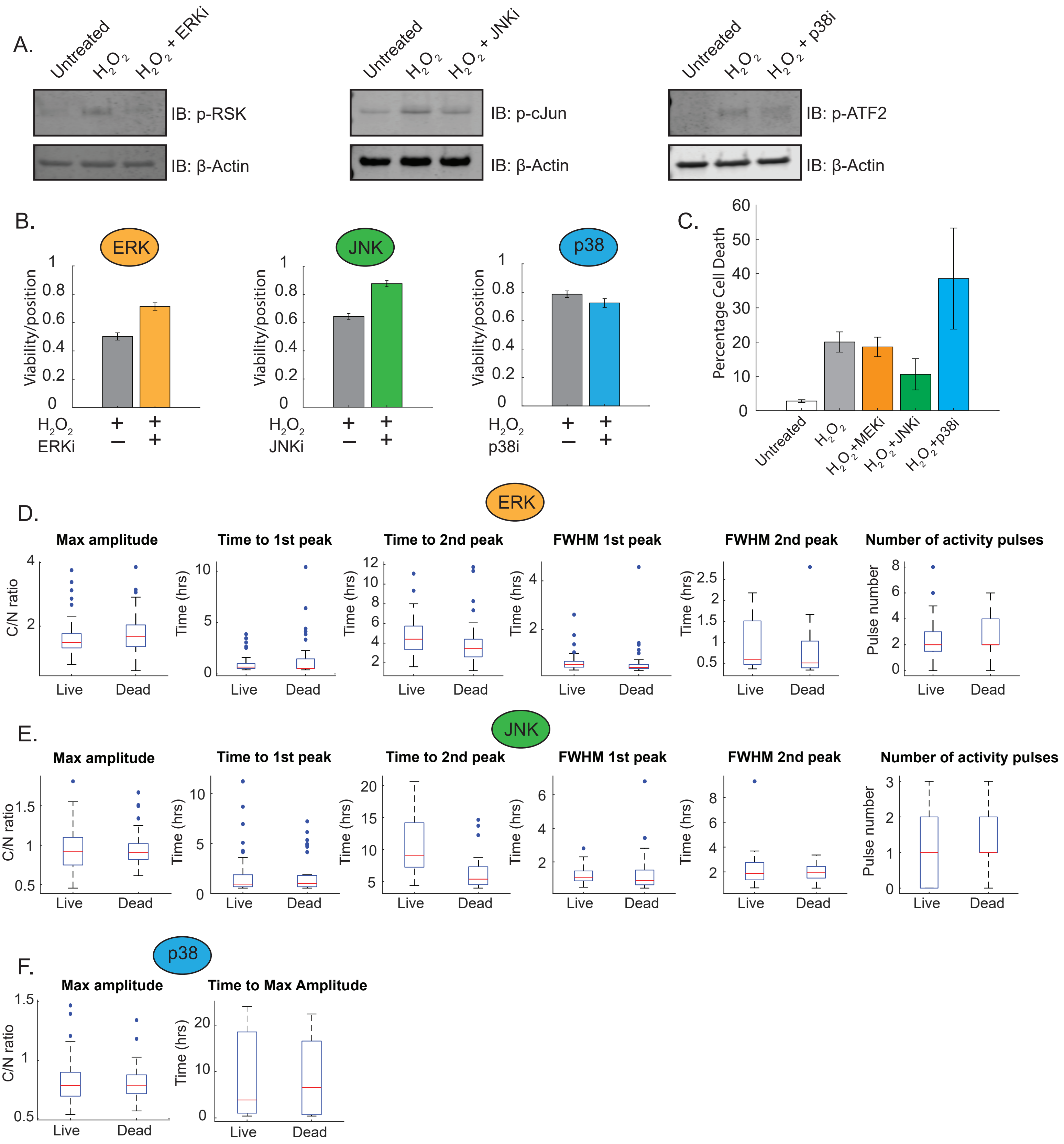

Appendix Figure S3. Impact of ERK, JNK, and p38 on H_2O_2-induced cell death, related to Figure 3.
A) Western blot analysis of downstream pathways regulated by ERK (panel 1), JNK (panel 2), and p38 (panel 3) confirming Ravoxertinib (ERKi), Tanzisertib (JNKi), and FHPI (p38i) block phosphorylation of downstream pathways.
B) Viability of cells in response to H_2O_2 in the presence or absence of ERKi, JNKi, or p38i as measured from imaging experiments. C) Percentage of cell death 24-hours post-treatment with 300 μM H_2O_2 in the presence or absence of U0126 (MEKi), SP600125 (JNKi), or Ralimetinib (p38i). Results based on three biological replicates (n=3)
D-F) Quantification of multiple dynamic features of kinase activation as measured in individual cells using KTR biosensors for ERK (D), JNK (E), and p38 (F). Data compares cells based on outcome in response to H_2O_2 treatment. Number of total cells analyzed in shown in Figure 3.

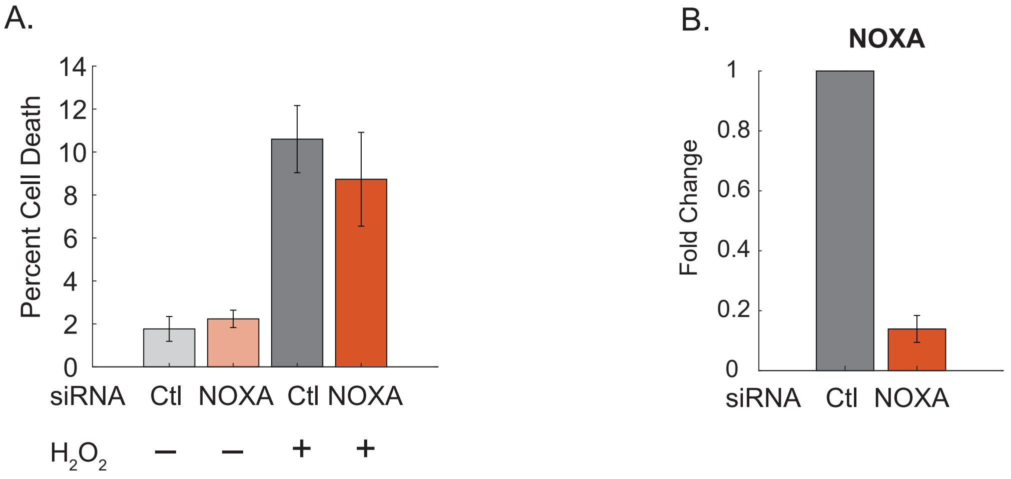

Appendix Figure S4. Knockdown of NOXA modestly reduces H_2O_2-induced cell death , related to Figure 4. A) Percentage of cell death observed in response to 300 μM H_2O_2 treatment in the presence or absence of NOXA knockdown. Data represents the mean±SEM of three biological replicates (n=3) B) RT-PCR analysis of NOXA expression following siRNA knockdown in HPNE cells. Data shown as Fold Change relative to control cells receiving non-targeting control. Data represents the mean±SEM of three biological replicates (n=3).

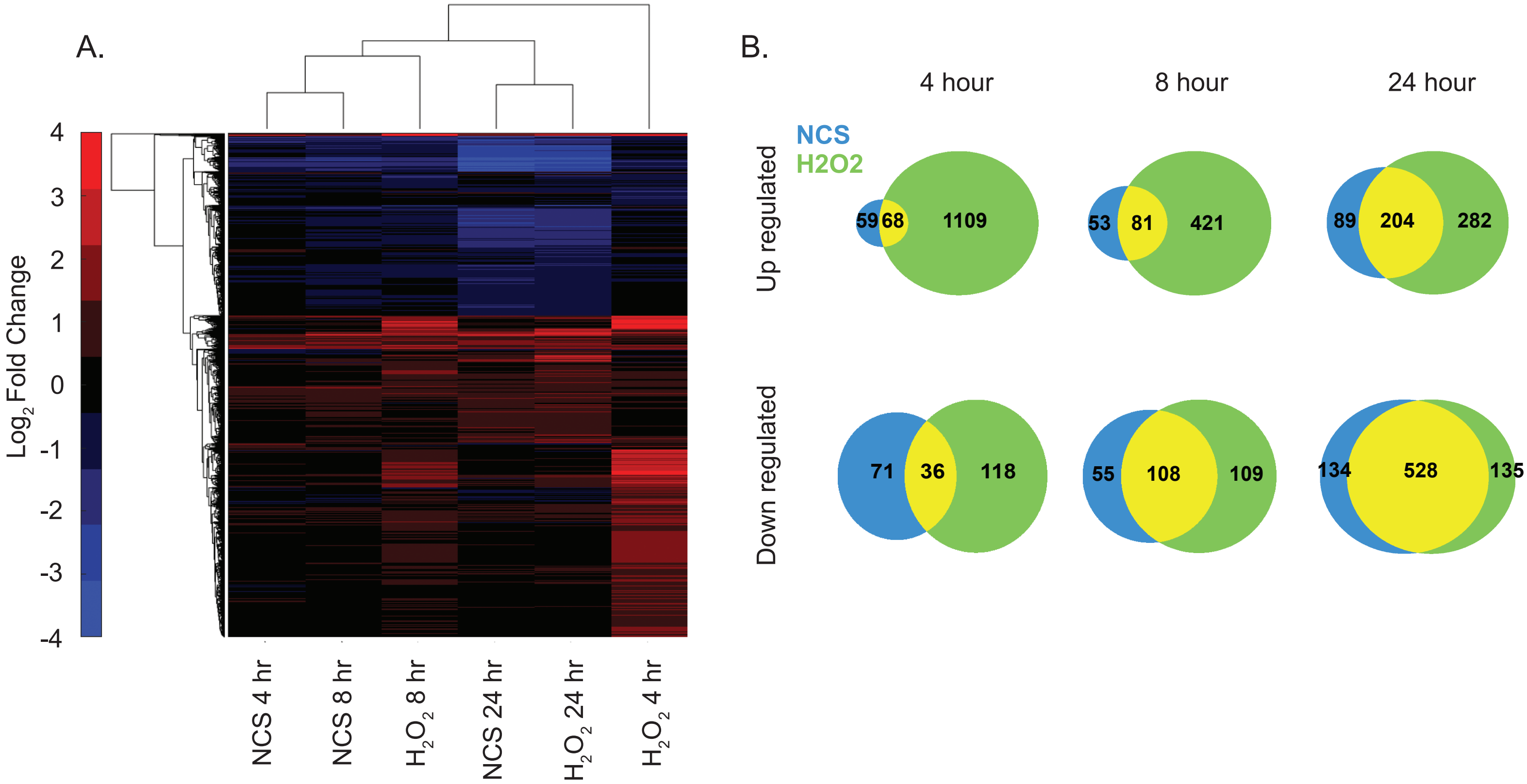

Appendix Figure S5. Late gene expression patterns of NCS and H_2O_2 are similar, related to Figure 5. A) Hierarchical clustering of differentially expressed genes between NCS and H_2O_2 treated cells. Color bar shows the scaling of the heatmap. At 24-hours the NCS and H_2O_2 treated samples cluster together. B) Venn diagrams illustrating the number of unique and shared up- and down-regulated genes in response to NCS or H_2O_2 over time. NCS and H_2O_2 show increasingly shared expression of up- and down-regulated genes at later time points.
