# Supplementary material for: Coordination of MAPK and p53 dynamics in the cellular responses to DNA damage and oxidative stress
Source: Mol Syst Biol. 2022 Dec 6;18(12):e11401. doi: 10.15252/msb.202211401 (PMC9724178; doi:10.15252/msb.202211401)
Supplement: Supplementary file 8 [file MSB-18-e11401-s002.pdf]

molecular systems biology

Coordination of MAPK and p53 dynamics in the cellular responses to DNA damage and oxidative stress

Ryan Hanson and Eric Batchelor
DOI: 10.15252/msb.202211401

Corresponding author(s): Eric Batchelor (ebatchel@umn.edu)

Review Timeline:

Submission Date:	14th Oct 22
Editorial Decision:	21st Oct 22
Revision Received:	1st Nov 22
Accepted:	7th Nov 22

Editor: Maria Polychronidou

Transaction Report:

(Note: With the exception of the correction of typographical or spelling errors that could be a source of ambiguity, letters and reports are not edited. Depending on transfer agreements, referee reports obtained elsewhere may or may not be included in this compilation. Referee reports are anonymous unless the Referee chooses to sign their reports.)

The reviewers' comments and authors' responses are not available with this article, as the initial review process took place with another journal.

1st Editorial Decision 21st Oct 2022

Thank you once again for submitting your manuscript "The dynamics of MAPK activity and p53 levels are coordinated to generate specificity in the cellular responses to DNA damage and oxidative stress" to Molecular Systems Biology. I have discussed with the team the manuscript and the reviewers' comments from the other journal. We think that the topic fits well to the scope of MSB. In line with the reviewers' comments, we acknowledge that the findings seem relevant for the field. We would be happy to publish the study in Molecular Systems Biology, pending some minor modifications and editorial issues that would need to be fixed.

Overall, we think that the concerns of the reviewers from the initial submission at the other journal have been satisfactorily addressed. Indeed, it seems that the remaining concern of reviewer #2 regarding the conceptual issue related to the dependence (or lack thereof, as the reviewer is concerned) of cell fates/responses on p53 seems to be the result of a misunderstanding. As such, we do not think that this concern prevents the publication of the study. Reviewer #2 also has two remaining technical concerns. We think that the first one, referring to the need to complement the results showing the effect of the chemical inhibitors by additional perturbations of the target genes, does not seem essential to address at this point. Regarding the second one, i.e. requesting validations of the apoptosis findings with additional apoptosis assays, we think that this is a good suggestion that indeed would strengthen the related findings. However, if you do not have such data at hand, or if it is very difficult to obtain them in a reasonable time frame, it seems reasonable to omit these additional analyses. Overall, we think that both remaining technical concerns of reviewer #2 can be addressed by some rather minor text modifications.

On a more editorial level, we would ask you to address the following points:

Response to Reviewers' comments and identification of erratum to original data

Dear Dr. Polychronidou,

We have addressed the reviewer 2's remaining comment regarding the quantification of apoptosis following the guidelines you suggested through text changes.

Page 7, line 7: Replaced the phrase "activating apoptosis" with "inducing cell death"

Page 8, line 4: In the Discussion, we included the statement: "While Annexin V and propidium iodide staining is typically indicative of apoptosis, other mechanisms of cell death are possible and further study is needed to elucidate the precise mechanism of cell death."

In the process of preparing the original data and analysis code for submission to GitHub, we noticed that some incorrect data had erroneously been included in some of the single cell data sets analyzed. Thankfully, correcting for the oversights did not alter any of the conclusions of the manuscript.

Specifically, in the third plot of Figure 1C, an additional 10 fluorescence traces had been mistakenly included in the H_2O_2 data set. We have corrected the number of samples in this figure from n = 55 (original plot) to n = 45 (corrected plot). This did not noticeably alter the associated third plot in Fig. 1C.

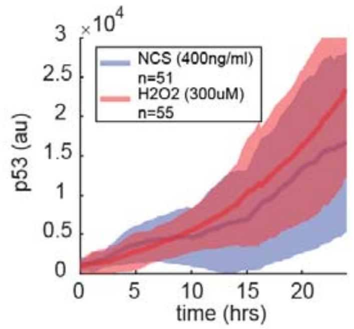

Original Fig. 1C

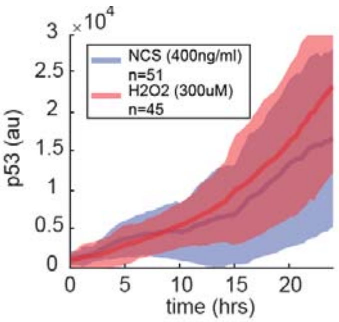

Corrected Fig. 1C

In Figure 1D, we provided statistical analysis of the data from Fig. 1C. Analysis of the corrected data set supports our original conclusion that there is not a statistically significant difference between the integrated p53 levels in the high DNA damage and the high H_2O_2 conditions, (p-values changing from p=0.27 to p=0.13 in the third set of bars, which is still not statistically significant).

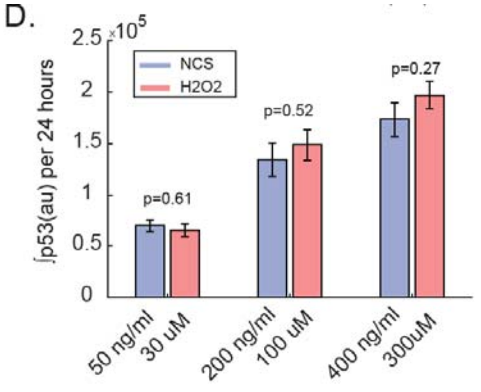

Original Fig. 1D

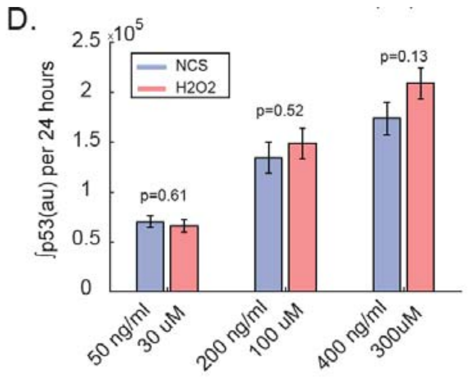

Corrected Fig. 1D

A similar error occurred in Fig. 2C, in which one erroneous trace was included in the H_2O_2 data set of ERK activity. We have removed the incorrectly included sample, and we updated the number of samples from n=62 to n=61. There was no appreciable effect on the figures, analysis, or conclusions drawn upon removal of the single sample.

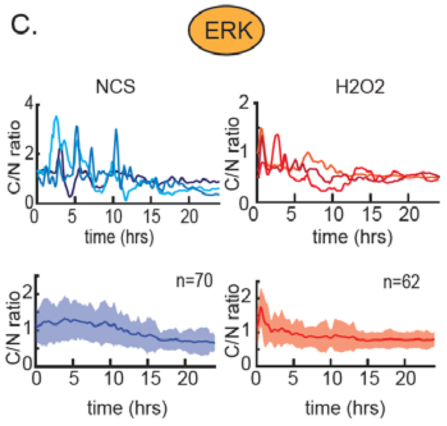

Original Fig. 2C

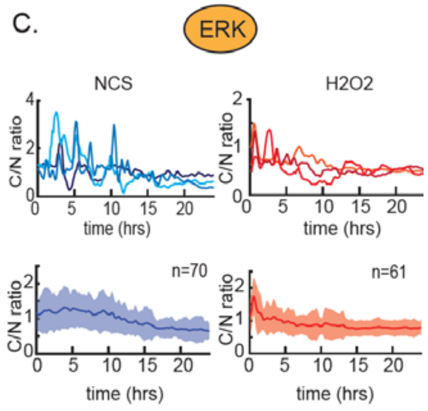

Corrected Fig. 2C

Data were also incorrectly included for the control condition in the experiment involving JNK inhibition, as presented in Fig. 6B. We have updated the figure to reflect the analysis of n=45 cells, accordingly. This resulted in the control bar having a slightly higher value (shown below). For the statistical analysis of these results, the corrected data actually provides greater statistical support for our conclusion that there is a significant difference between the control cells and the cells treated with the JNK inhibitor, with the p-value changing from 0.013 to 0.005. The correct p-value has now been reported in the caption for Figure 6B.

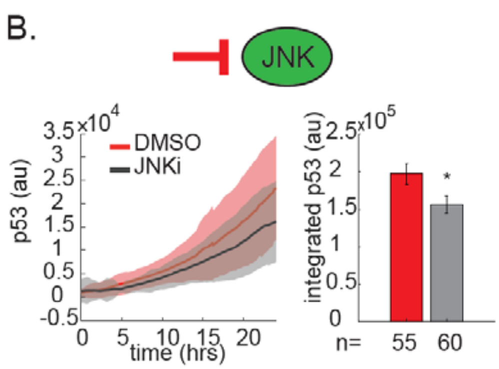

Original Fig. 6B

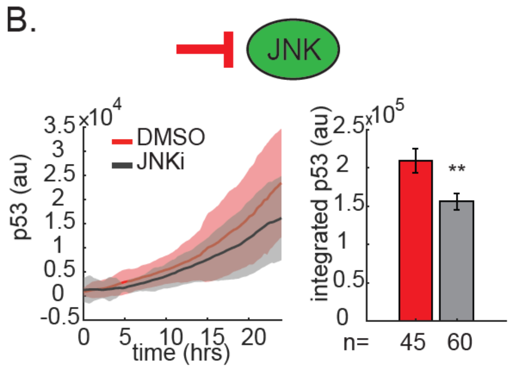

Corrected Fig. 6B

We apologize for the errors, which fortunately have been caught at this stage and which have not altered any of the conclusions of the manuscript.

1st Revision - Editorial Decision 7th Nov 2022

Thank you again for sending us your revised manuscript. We are now satisfied with the modifications made and I am pleased to inform you that your paper has been accepted for publication.

EMBO Press Author Checklist

Corresponding Author Name: Eric Batchelor	
Journal Submitted to: Molecular Systems Biology	
Manuscript Number: MSB-2022-11401R	

USEFUL LINKS FOR COMPLETING THIS FORM
[The EMBO Journal - Author Guidelines]
[EMBO Reports - Author Guidelines]
[Molecular Systems Biology - Author Guidelines]
[EMBO Molecular Medicine - Author Guidelines]

Reporting Checklist for Life Science Articles (updated January
This checklist is adapted from Materials Design Analysis Reporting (MDAR) Checklist for Authors. MDAR establishes a minimum set of requirements in transparent reporting in the life sciences (see Statement of Task: 10.31222/osf.io/9sm4x). Please follow the journal's guidelines in preparing your
Please note that a copy of this checklist will be published alongside your article.

Abridged guidelines for figures
1. Data
The data shown in figures should satisfy the following conditions:
- the data were obtained and processed according to the field's best practice and are presented to reflect the results of the experiments in an accurate and unbiased manner.
- ideally, figure panels should include only measurements that are directly comparable to each other and obtained with the same assay.
- plots include clearly labeled error bars for independent experiments and sample sizes. Unless justified, error bars should not be shown for technical
- if n<5, the individual data points from each experiment should be plotted. Any statistical test employed should be justified.
- Source Data should be included to report the data underlying figures according to the guidelines set out in the authorship guidelines on Data

2. Captions
Each figure caption should contain the following information, for each panel where they are relevant:
- a specification of the experimental system investigated (eg cell line, species name).
- the assay(s) and method(s) used to carry out the reported observations and measurements.
- an explicit mention of the biological and chemical entity(ies) that are being measured.
- an explicit mention of the biological and chemical entity(ies) that are altered/varied/perturbed in a controlled manner.
- the exact sample size (n) for each experimental group/condition, given as a number, not a range;
- a description of the sample collection allowing the reader to understand whether the samples represent technical or biological replicates (including how many animals, litters, cultures, etc.).
- a statement of how many times the experiment shown was independently replicated in the laboratory.
- definitions of statistical methods and measures:
  - common tests, such as t-test (please specify whether paired vs. unpaired), simple χ2 tests, Wilcoxon and Mann-Whitney tests, can be unambiguously identified by name only, but more complex techniques should be described in the methods section;
  - are tests one-sided or two-sided?
  - are there adjustments for multiple comparisons?
  - exact statistical test results, e.g., P values = x but not P values < x;
  - definition of 'center values' as median or average;
  - definition of error bars as s.d. or s.e.m.

Please complete ALL of the questions below.
Select "Not Applicable" only when the requested information is not relevant for your study.

Materials

Newly Created Materials	Information included in the manuscript?	In which section is the information available? (Reagents and Tools Table, Materials and Methods, Figures, Data Availability Section)
New materials and reagents need to be available; do any restrictions apply?	Yes	Resource Availability sections

Antibodies	Information included in the manuscript?	In which section is the information available? (Reagents and Tools Table, Materials and Methods, Figures, Data Availability Section)
For **antibodies** provide the following information:<br>- Commercial antibodies: RRID (if possible) or supplier name, catalogue number and or/clone number<br>- Non-commercial: RRID or citation	Yes	Materials and Methods

DNA and RNA sequences	Information included in the manuscript?	In which section is the information available? (Reagents and Tools Table, Materials and Methods, Figures, Data Availability Section)
**Short novel DNA or RNA including primers, probes**: provide the sequences.	Yes	Materials and Methods

Cell materials	Information included in the manuscript?	In which section is the information available? (Reagents and Tools Table, Materials and Methods, Figures, Data Availability Section)
**Cell lines:** Provide species information, strain. Provide accession number in repository **OR** supplier name, catalog number, clone number, and/**OR** RRID.	Yes	Materials and Methods
**Primary cultures:** Provide species, strain, sex of origin, genetic modification status.	Not Applicable	
Report if the cell lines were recently **authenticated** (e.g., by STR profiling) and tested for mycoplasma contamination.	Yes	Materials and Methods

Experimental animals	Information included in the manuscript?	In which section is the information available? (Reagents and Tools Table, Materials and Methods, Figures, Data Availability Section)
**Laboratory animals or Model organisms:** Provide species, strain, sex, age, genetic modification status. Provide accession number in repository **OR** supplier name, catalog number, clone number, **OR** RRID.	Not Applicable	
**Animal observed in or captured from the field:** Provide species, sex, and age where possible.	Not Applicable	
Please detail **housing and husbandry conditions**.	Not Applicable	

Plants and microbes	Information included in the manuscript?	In which section is the information available? (Reagents and Tools Table, Materials and Methods, Figures, Data Availability Section)
**Plants:** provide species and strain, ecotype and cultivar where relevant, unique accession number if available, and source (including location for collected wild specimens).	Not Applicable	
**Microbes:** provide species and strain, unique accession number if available, and source.	Not Applicable	

Human research participants	Information included in the manuscript?	In which section is the information available? (Reagents and Tools Table, Materials and Methods, Figures, Data Availability Section)
If collected and within the bounds of privacy constraints report on age, sex and gender or ethnicity for all study participants.	Not Applicable	

Core facilities	Information included in the manuscript?	In which section is the information available? (Reagents and Tools Table, Materials and Methods, Figures, Data Availability Section)
If your work benefited from core facilities, was their service mentioned in the acknowledgments section?	Yes	Acknowledgements

Design

Study protocol	Information included in the manuscript?	In which section is the information available? (Reagents and Tools Table, Materials and Methods, Figures, Data Availability Section)
If study protocol has been **pre-registered, provide DOI in the manuscript**. For clinical trials, provide the trial registration number **OR** cite DOI.	Not Applicable	
Report the **clinical trial registration number** (at ClinicalTrials.gov or equivalent), where applicable.	Not Applicable	

Laboratory protocol	Information included in the manuscript?	In which section is the information available? (Reagents and Tools Table, Materials and Methods, Figures, Data Availability Section)
Provide DOI OR other citation details if **external detailed step-by-step protocols** are available.	Not Applicable	

Experimental study design and statistics	Information included in the manuscript?	In which section is the information available? (Reagents and Tools Table, Materials and Methods, Figures, Data Availability Section)
Include a statement about **sample size** estimate even if no statistical methods were used.	Yes	Figures
Were any steps taken to minimize the effects of subjective bias when allocating animals/samples to treatment (e.g. **randomization procedure**)? If yes, have they been described?	Not Applicable	
Include a statement about **blinding** even if no blinding was done.	Not Applicable	
Describe **inclusion/exclusion criteria** if samples or animals were excluded from the analysis. Were the criteria pre-established?<br><br>If sample or data points were omitted from analysis, report if this was due to attrition or intentional exclusion and provide justification.	Not Applicable	
For every figure, are **statistical tests** justified as appropriate? Do the data meet the assumptions of the tests (e.g., normal distribution)? Describe any methods used to assess it. Is there an estimate of variation within each group of data? Is the variance similar between the groups that are being statistically compared?	Yes	Figures

Sample definition and in-laboratory replication	Information included in the manuscript?	In which section is the information available? (Reagents and Tools Table, Materials and Methods, Figures, Data Availability Section)
In the figure legends: state number of times the experiment was **replicated** in laboratory.	Yes	Figures
In the figure legends: define whether data describe **technical or biological replicates**.	Yes	Figures

Ethics

Ethics	Information included in the manuscript?	In which section is the information available? (Reagents and Tools Table, Materials and Methods, Figures, Data Availability Section)
Studies involving **human participants**: State details of **authority granting ethics approval** (IRB or equivalent committee(s), provide reference number for approval.	Not Applicable	
Studies involving **human participants**: Include a statement confirming that **informed consent** was obtained from all subjects and that the experiments conformed to the principles set out in the WMA Declaration of Helsinki and the Department of Health and Human Services Belmont Report.	Not Applicable	
Studies involving **human participants:** For publication of **patient photos**, include a statement confirming that consent to publish was obtained.	Not Applicable	
Studies involving experimental **animals**: State details of **authority granting ethics approva**l (IRB or equivalent committee(s), provide reference number for approval. Include a statement of compliance with ethical regulations.	Not Applicable	
Studies involving **specimen and field samples:** State if relevant **permits** obtained, provide details of authority approving study; if none were required, explain why.	Not Applicable	

Dual Use Research of Concern (DURC)	Information included in the manuscript?	In which section is the information available? (Reagents and Tools Table, Materials and Methods, Figures, Data Availability Section)
Could your study fall under dual use research restrictions? Please check biosecurity documents and list of **select agents and toxins** (CDC): https://www.selectagents.gov/sat/list.htm	Not Applicable	
If you used a select agent, is the security level of the lab appropriate and reported in the manuscript?	Not Applicable	
If a study is subject to dual use research of concern regulations, is the name of the **authority granting approval and reference number** for the regulatory approval provided in the manuscript?	Not Applicable	

Reporting

The MDAR framework recommends adoption of discipline-specific guidelines, established and endorsed through community initiatives. Journals have their own policy about requiring specific guidelines and recommendations to complement MDAR.

Adherence to community standards	Information included in the manuscript?	In which section is the information available? (Reagents and Tools Table, Materials and Methods, Figures, Data Availability Section)
State if relevant guidelines or checklists (e.g., **ICMJE, MIBBI, ARRIVE, PRISMA**) have been followed or provided.	Not Applicable	
For **tumor marker prognostic studies,** we recommend that you follow the **REMARK** reporting guidelines (see link list at top right). See author guidelines, under 'Reporting Guidelines'. Please confirm you have followed these guidelines.	Not Applicable	
For **phase II and III randomized controlled trials**, please refer to the **CONSORT** flow diagram (see link list at top right) and submit the CONSORT checklist (see link list at top right) with your submission. See author guidelines, under 'Reporting Guidelines'. Please confirm you have submitted this list.	Not Applicable	

Data Availability

Data availability	Information included in the manuscript?	In which section is the information available? (Reagents and Tools Table, Materials and Methods, Figures, Data Availability Section)
Have **primary datasets** been deposited according to the journal's guidelines (see 'Data Deposition' section) and the respective accession numbers provided in the Data Availability Section?	Yes	Data Availablility section
Were **human clinical and genomic datasets** deposited in a public access-controlled repository in accordance to ethical obligations to the patients and to the applicable consent agreement?	Not Applicable	
Are **computational models** that are central and integral to a study available without restrictions in a machine-readable form? Were the relevant accession numbers or links provided?	Yes	Data Availablility section
If publicly available data were reused, provide the respective **data citations in the reference list**.	Not Applicable	
